# A continuous flow process for the Ireland–Claisen rearrangement

**DOI:** 10.1039/d5ra07248d

**Published:** 2025-11-24

**Authors:** Joseph D'Attoma, Stéphane Bostyn, Sylvain Routier, Karen Plé, Frédéric Buron

**Affiliations:** a Université D’Orléans, CNRS, ICOA, UMR 7311 France karen.ple@univ-orleans.fr frederic.buron@univ-orleans.fr; b ICARE, CNRS, UPR 45071 Cedex 2 Orléans 3021 France

## Abstract

Herein, we describe an in-depth study of an enantioselective Ireland–Claisen rearrangement using a continuous flow reactor. The use of this innovative technology led to a rapid reaction at room temperature, enabling the stereocontrolled synthesis of vicinal stereogenic centers with little to no loss in yield or optical purity, in the absence of cryogenic conditions. The reaction scope was examined *via* the synthesis of several allyl esters, and the results were discussed. The validation and scale-up of the process were also performed.

## Abstract 2

The transposition of an enantioselective Ireland–Claisen rearrangement was performed in a continuous flow reactor with a short residence time, achieving great stereocontrolled syntheses of vicinal stereogenic centers at room temperature.

## Graphical abstract

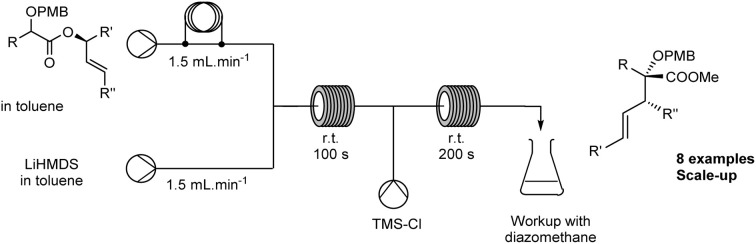

## Introduction

In the past decade, flow chemistry has become an emerging tool in organic synthesis due to its significant improvements in scalability, energy efficiency, safety, access to a wider range of reaction conditions, and unique opportunities in multi-step synthesis.^1–10^ Further development of the continuous process is identified as one of the most important areas of research in green chemistry^11–13^ and engineering for the pharmaceutical industry.^14–20^ The main advantage of flow chemistry is the use of low reaction mixture volumes, coupled with a high surface area to volume ratio, which allows a greater heat exchange control. Highly exothermic reactions, that would otherwise need cryogenic conditions, can be operated at higher reaction temperatures than those possible under batch conditions, such as anionic reactions, nitration, or alkene ozonolysis.^21–24^

**Scheme 1:**
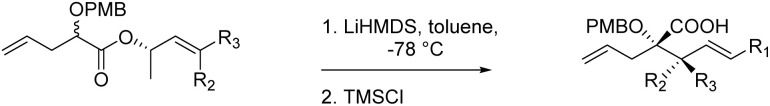
Cryogenic Claisen Ireland rearrangement.

Rearrangement reactions have also been impacted by the need to find safer and more sustainable procedures, especially for the production of pharmaceutically relevant scaffolds that are difficult to be synthesized by other methods.^25^ In this context, the Ireland–Claisen rearrangement has been proven to be an essential procedure for the stereoselective formation of carbon–carbon bonds by the conversion of allyl esters to γ,δ-unsaturated carboxylic acids.^26–30^ Its key step is a [3,3]-sigmatropic rearrangement of a silyl ketene acetal, which is generated *in situ* by deprotonation of an allyl ester using a strong base. This rearrangement has been widely used in natural product synthesis^31,32^ and has also been shown to be extremely useful in the formation of quaternary stereogenic centers.^33,34^ In general, the required allylic esters are easily accessible from carboxylic acids and allylic alcohols. Moreover, when a chiral substituted allylic alcohol is used, the asymmetric configuration of the alcohol and the double bond geometry offer a controlled access to any one of the four possible stereoisomers. As a result, the Ireland–Claisen rearrangement proceeds with a high degree of stereoselectivity due to the chirality transfer from the carbinol center to the newly generated stereocenter(s). When an additional substituent is present at the alpha position to the ester function, two adjacent quaternary carbons are generated in one step (Scheme 1). We have previously demonstrated that a Claisen rearrangement/metathesis sequence can be applied to the enantio- and diastereo-selective synthesis of quaternary hydroxy and amino acid carbocycles from allylic esters.^35^

Enolate formation in the Ireland–Claisen rearrangement is typically performed at a low temperature (−78 °C) in the presence of a strong base (LDA or LiHMDS), and the rearrangement takes place while warming the reaction mixture for several hours. These conditions work well on a small scale but are not easily amenable to large scale reactions. As part of our on-going research in flow chemistry, we were interested in adapting this rearrangement from batch to flow. We thus embarked on a more comprehensive study for the adaptation of the Ireland–Claisen reaction into a continuous flow process. The scope and limitations of the process were explored using conventional allylic esters. The challenges encountered during the investigation as well as in the scale-up phase are disclosed in this study.

## Results and discussion

Typically, standard rearrangement reactions employ a hindered non-nucleophilic strong base for the efficient low temperature enolization of the allylic ester followed by the silylation of the ester enolate to avoid side reactions such as decomposition *via* the ketene pathway or aldol type condensations. The continuous-flow process appeared to us as a method of choice to tackle the formation of the highly reactive and sensitive organolithium species by finely adjusting the residence time and temperature of the reactor, which are the two crucial parameters in organolithium chemistry. At the beginning of this study, we selected the known ester 1 as a model substrate to study the rearrangement as the obtained diastereoisomers were both characterized and differentiated by NMR.^36^ We began by optimizing the enolate formation using LiHMDS at room temperature in a cascade procedure, as shown in Scheme 1. The enolate was trapped by using an excess of TMSCl, followed by a rapid rearrangement while warming to room temperature. Extraction and treatment of the crude acid by a diazomethane solution^37^ gave the corresponding ester and allowed us to easily calculate the yield and diastereoselectivity of the rearrangement product (Table 1).

**Table 1:** Optimization of Ireland–Claisen reaction under a semi-continuous processing mode

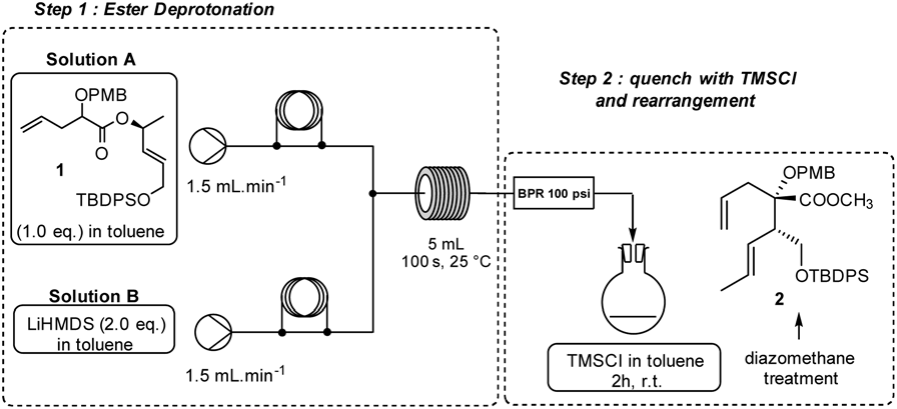					
Entry	Coil volume (mL)	Temp (°C)	Resident time (sec)	Yield^a^ (%)	Diastereomeric ratio^b^
1	10	22 °C	200	10	85/15
2	2.5	22 °C	50	64	85/15
3	2.5	0 °C	50	39	87/13
4	5	22 °C	100	70	87/13
5	5	40 °C	100	72	83/17

a Isolated yield.

b Diastereomeric ratio (dr) was calculated by ^1^H NMR on the purified compounds.

To perform the reaction, solution A containing 1 (0.358 mmol) in toluene (2 mL) was introduced at a flow rate of 1.5 mL min^−1^ and mixed using a standard T-junction with a second solution B containing freshly prepared LiHMDS (0.716 mmol, 2.0 equiv.) in toluene (2 mL) and pumped at the same flow rate. This combined solution was then passed through a tubular reactor (1 mm internal diameter, PTFE). The reaction was carried out repeatedly while varying the residence time and temperature. As provided in Table 1, a long residence time at room temperature was detrimental to the reaction (Table 1, entry 1), presumably because of the decomposition of the anionic intermediate. However, compound 2 was isolated in 10% yield with a high diastereoselectivity (diastereomeric ratio (dr): 85/15 *vs.* 61%, 80/20 dr in classical batch conditions: 6 h, −78 °C to 0 °C). At a short residence time, the yield increased significantly to 64% with the same 85/15 dr (Table 1, entry 2). A reduction in the temperature resulted in a dramatic decrease in yield because deprotonation was incomplete at a lower temperature with a short residence time (Table 1, entry 3). The best result was achieved with a residence time of 100 seconds at room temperature, obtaining 2 in a good yield (70%, 87/13 dr) (Table 1, entry 4). Increasing the temperature to 40 °C did not affect the efficiency of the reaction but led to a slight decrease in diastereoselectivity (Table 1, entry 5).

**Fig. 1:**
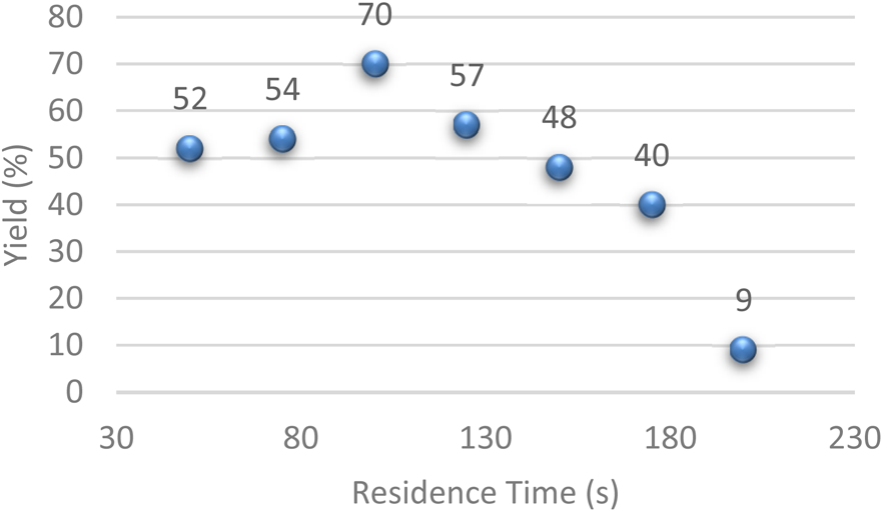
Evolution of yield *vs.* resident time at r.t.

In order to demonstrate the importance of residence time on enolate formation and consequently on the reaction yield, we followed its evolution as a function of the residence time at room temperature. The obtained result showed that the residence time was of crucial importance, and optimal enolate formation was reached at 100 seconds. Before 100 s, we assume that enolate was not totally formed and that after 100 s, degradation took place (Fig. 1).

**Fig. 2:**
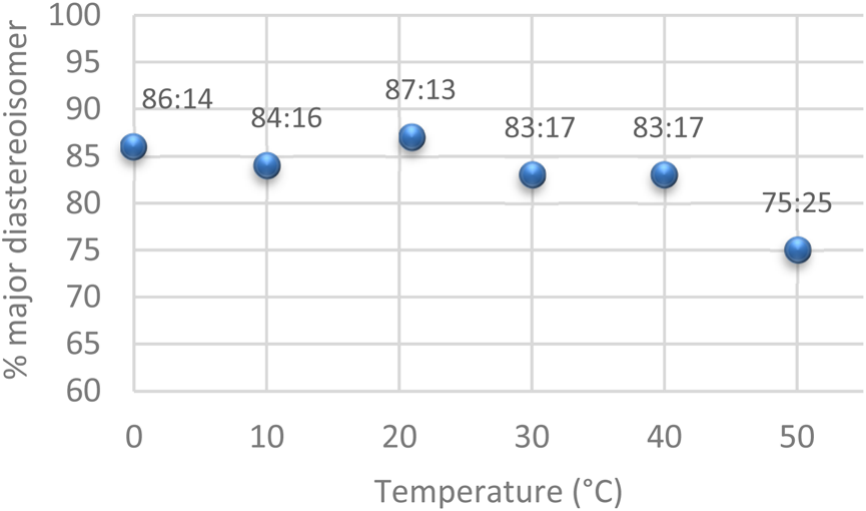
Influence of temperature on diastereomeric ratio with a residence time of 100 seconds.

After identifying the optimal residence time, a similar study was carried out to establish the effect of temperature on the diastereomeric ratio. As shown in Fig. 2, temperature had little to no impact on the dr, remaining quasi-stable from 0 °C up to 40 °C. In this temperature range, the six-member chair-like transition state *via* the formation of the (*Z*)-enolate was favoured, while above 40 °C, the (*E*) enolate form is more predominant, leading to a reduced diastereomeric ratio.

**Scheme 2:**
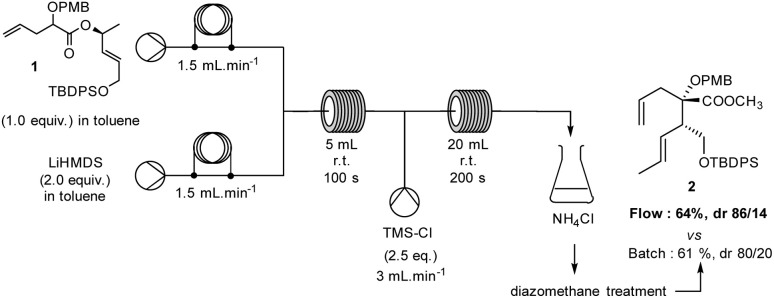
Ireland–Claisen Reaction in a continuous processing mode.

In order to create a fully continuous process, we decided to add a third pump, thus allowing us to trap the anion with TMSCl and induce a rapid rearrangement in a second flow reactor. A solution of TMSCl in toluene (2.5 equiv., solution C) was placed after reactor 1, using both a second standard T-junction and reactor (20 mL, 1 mm internal diameter, PTFE) (Scheme 2). With this modification, the holding time of reactor 2 was regulated to 200 seconds due to the total flow rate imposed by the sum of each individual throughput rate (solution A: 0.36 mol L^−1^, 1.5 mL min^−1^; solution B: 0.72 mol L^−1^, 1.5 mL min^−1^; solution C: 0.45 mol L^−1^, 3.0 mL min^−1^), resulting in a continuous process in a total holding time of 300 seconds. The desired product 2 was quenched with NH_4_Cl at the outlet of the reactor to terminate the reaction. The extracted product was then treated with diazomethane. With this online process, compound 2 was isolated in good yield and diastereoselectivity (64%, dr 86/14 *versus* Batch 61%, dr 80/20).

Then, with these optimized flow conditions in hand, we evaluated the scope of the reaction with several esters (Table 2). First, the substitution at the carbinol position with an α-isopropyl group 3 in the presence of only 2 equivalents of base produced the methyl ester 11 in 62% yield. The added steric hindrance of the isopropyl *vs.* methyl group increased the dr (>99%), but the reactivity diminished due to the recovery of the starting material. Carrying out the rearrangement with 3.0 equivalents of base increased the reaction yield to 59% without affecting the dr (>99%), producing only trace amounts of the starting material. A final reaction with 4.0 equiv. of LiHMDS increased the yield of 11 to 62% (dr > 99%) with no starting material detected. As a result, we decided to continue exploring our reaction scope in the presence of 4 equiv. of LiHMDS.

**Table 2:** Scope of Ireland–Claisen reaction

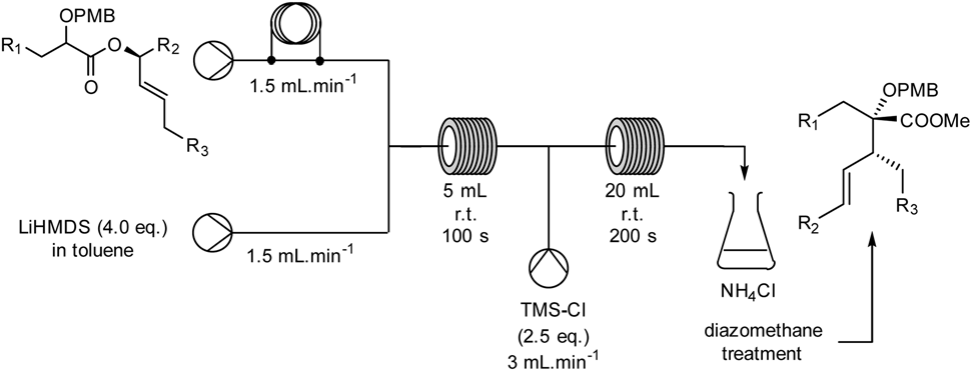		
Entry	Ester	Compound, yield^a^, dr^b^ or er^c^
1	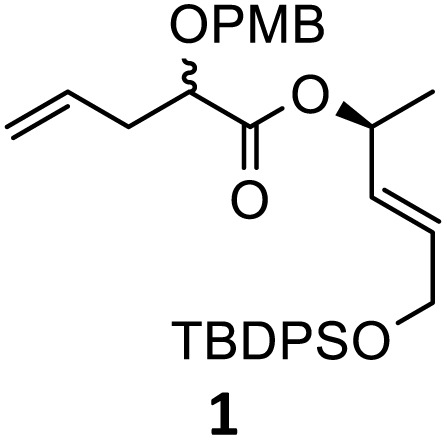	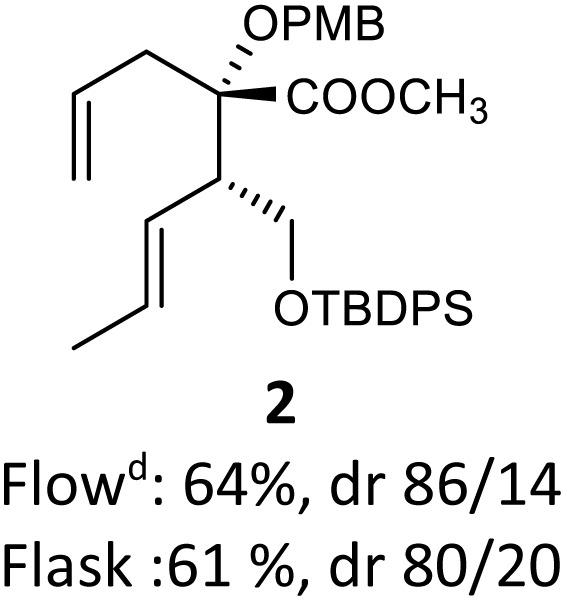
2	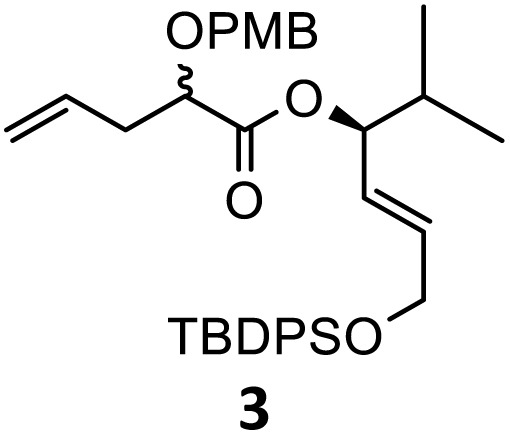	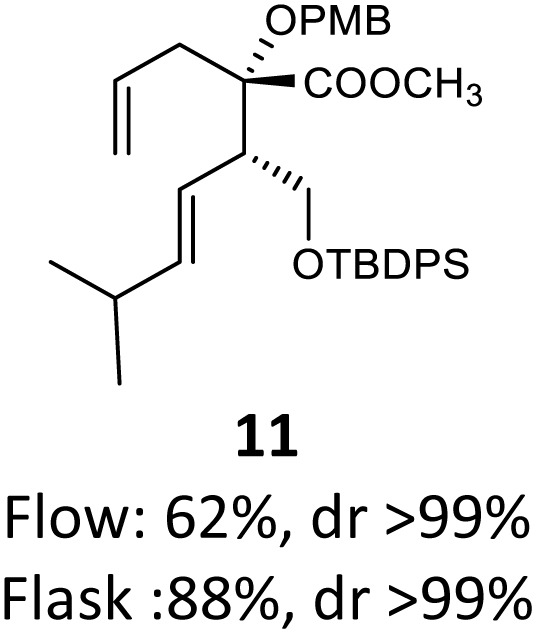
3	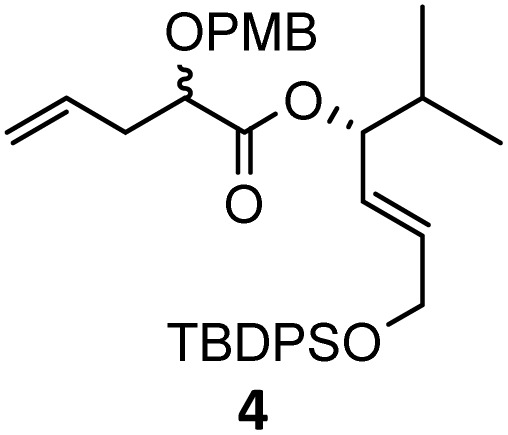	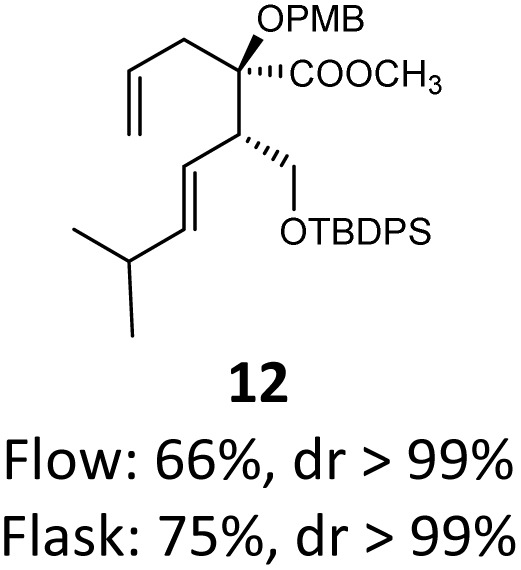
4	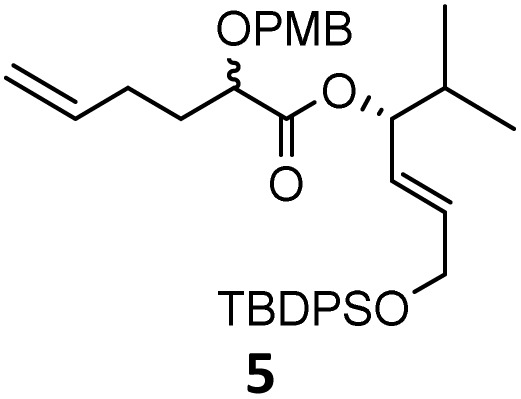	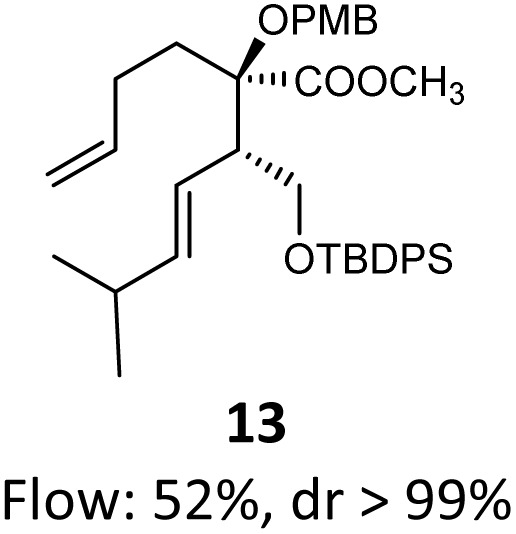
5	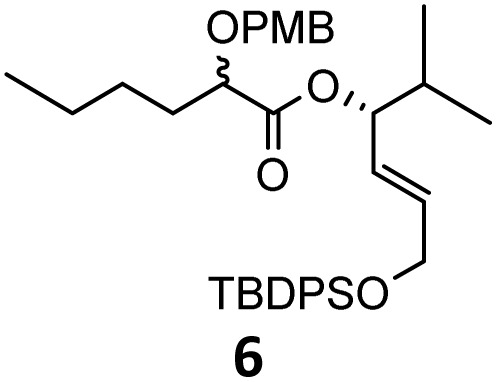	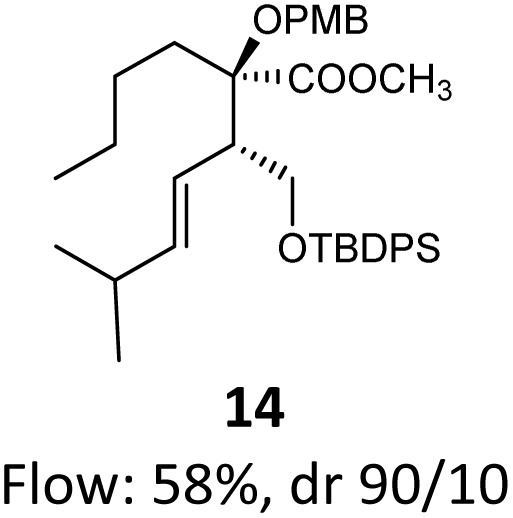
6	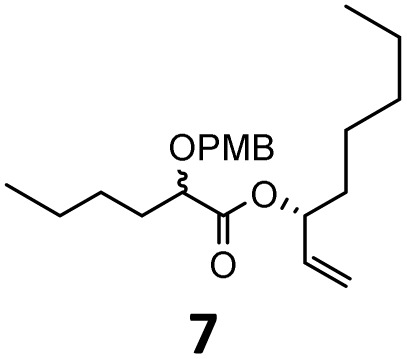	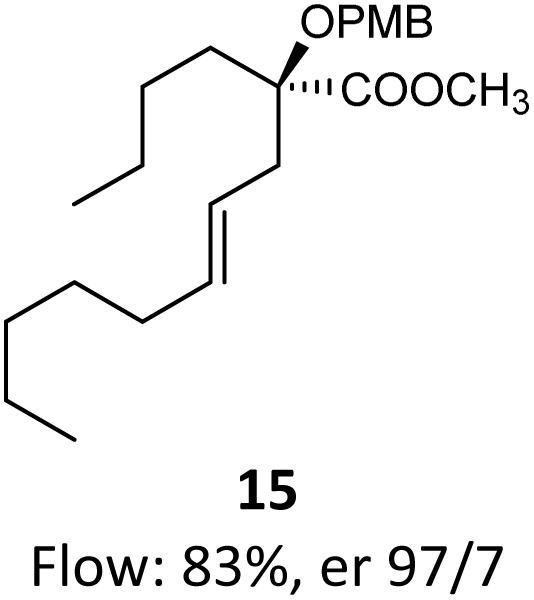
7	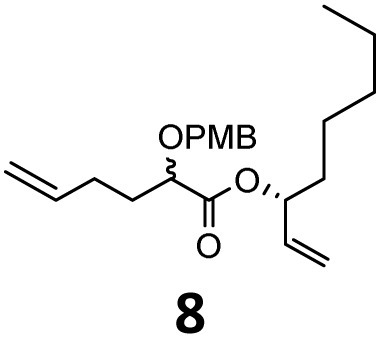	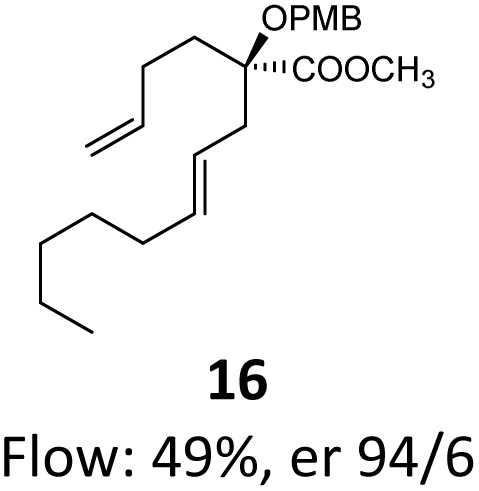
8	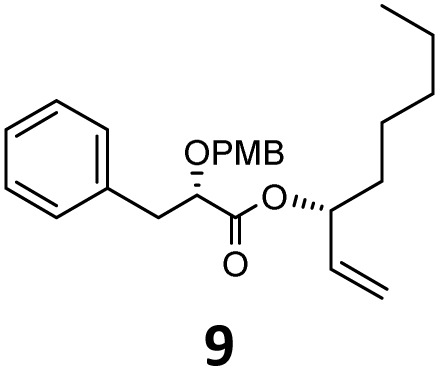	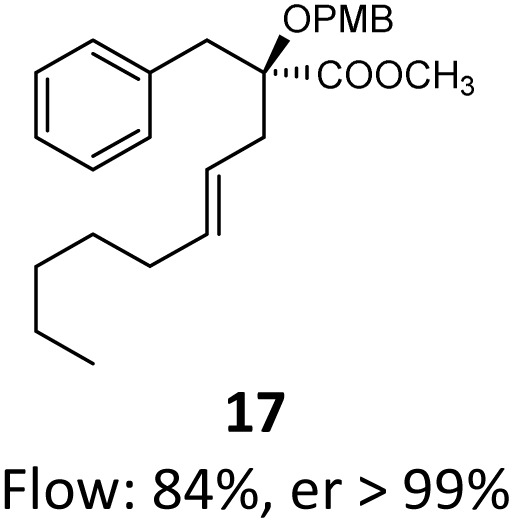

a Yields were determined on isolated products.

b Diastereomeric ratio (dr) was calculated by conducting ^1^H NMR on the purified compounds.

c Enantiomeric ratio (er) was measured by supercritical fluid chromatography (SFC) on the purified compounds.

d 2.0 equiv. of LiHMDS was used.

When these new conditions were applied to the stereoisomer 4, the same behavior was observed, giving the desired compound 12 in good yield and dr with the opposite configuration at the quaternary carbon center, showing an exceptional level of stereocontrol.^35^ The homologated ester 5 and the aliphatic ester 6 furnished the rearrangement products 13 and 14, respectively, with the same excellent dr accompanied by slightly lower yields (52% and 58%, respectively, *versus* 62% for 11). We next switched to the unsaturated ester 7 and the corresponding saturated ester 8, which were prepared from (*S*)-1-octen-3-ol to evaluate the enantioselective variant of the rearrangement. The reaction of the ester 7 proceeded with a high level of stereoselectivity and offered high yield (Table 2, entry 6), while the corresponding alkene derivative 8 gave the rearrangement product 16 in low yield but with a similar enantioselectivity (Table 2, entry 7). The rearrangement of the hydrocinnamic acid ester derivative 9 was also successful, providing the desired product 17 as a single enantiomer in 84% yield.

**Scheme 3:**
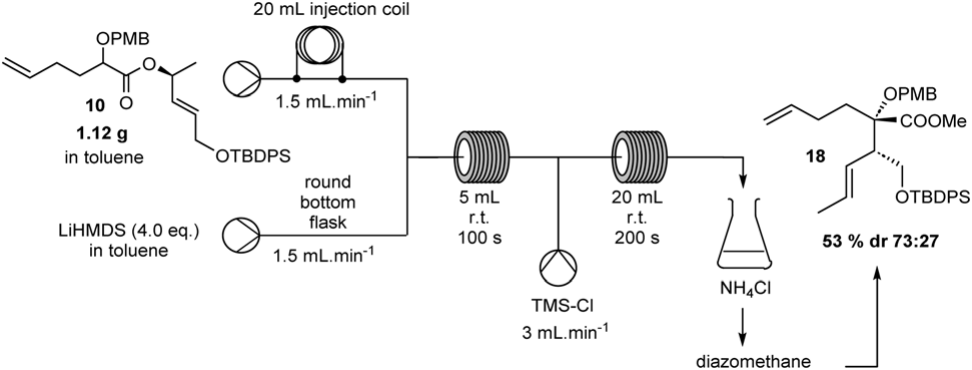
Scale-up of Ireland–Claisen reaction.

Finally, we demonstrated the synthetic interest of our methodology with a scale-up procedure (Scheme 3). As previously mentioned, the major drawback of the traditional Ireland–Claisen rearrangement and the use of lithiated species (in general) demand controlled cryogenic conditions (−78 °C). In order to validate our protocol in a scale-up reaction, we investigated the Ireland–Claisen rearrangement with a run time of 10 min (molar flow: 0.126 mmol min^−1^ for ester 10, 0.504 mmol min^−1^ for LiHMDS and 0.158 mmol min^−1^ for TMSCl) with our optimized flow conditions. We observed that the system was fully stabilized during the run time, and no exothermicity was observed in the two reactors. Interestingly, the throughput rate of the rearrangement product 18 was up to 2.09 g h^−1^ with only a moderately lower diastereoselectivity (dr 73/27, 53% yield). These findings suggest that this system could be very effective for designing a safe pilot Ireland–Claisen flow process in a single one-pot continuous sequence without using cryogenic conditions or hazardous materials.

## Conclusions

In conclusion, we developed an Ireland–Claisen rearrangement strategy under continuous-flow conditions. First, the development of an efficient semi-continuous process at room temperature was established with great efficiency and high level of stereoselectivity. In the second step, the implementation of a three-inlet flow setup made it possible to overcome the cascade step for silylation. This fully continuous process also demonstrated high levels of reactivity and stereoselectivity with a representative panel of allyl esters. Finally, the efficiency of the reaction was maintained over 10 min, without any observed exothermicity. We believe that this novel process will find applications in both academic and industrial settings and inspire further developments in sigmatropic reactions that involve lithium species.

## Author contributions

Karen Plé, Stéphane Bosytn, Sylvain Routier, Frédéric Buron: conceptualization, investigation, validation, methodology, resources, supervision, validation, writing-original draft, and editing. Joseph d’Attoma: validation, investigation.

## Conflicts of interest

There are no conflicts to declare.

## Supplementary Material

RA-015-D5RA07248D-s001

## Data Availability

The data underlying this study are available in the published article and its supplementary information (SI). Supplementary information: experimental procedures, product characterization, and ^1^H and ^13^C NMR spectra of the products. See DOI: https://doi.org/10.1039/d5ra07248d.
