# Supplementary material for: A continuous flow process for the Ireland–Claisen rearrangement
Source: RSC Adv. 2025 Nov 24;15(53):45719–23. doi: 10.1039/d5ra07248d (PMC12641246; doi:10.1039/d5ra07248d)
Supplement: RA-015-D5RA07248D-s001 [file RA-015-D5RA07248D-s001.pdf]

Supplementary Information (SI) for RSC Advances.
This journal is © The Royal Society of Chemistry 2025

A Continuous Flow Process for the Ireland-Claisen rearrangement

Joseph D'Attoma[a], Stéphane Bostyn[b], Sylvain Routier[a], Karen Plé[a,*] and Frédéric Buron[a,*]

a. Université d'Orléans, CNRS, ICOA, UMR 7311, France.
b. ICARE, CNRS UPR3021, 45071 Cedex 2 Orléans, France.

Table of Contents

General Information	2
Preparation of starting materials	2-5
General procedures	5-6
Synthesis of compounds **5**-**17**	6-12
References	12
^{1}H NMR, ^{13}C NMR	13-27

1- General informations

Chemicals and analytical grade solvents were purchased from commercial suppliers and used without further purification unless otherwise stated. Reactions requiring anhydrous conditions were performed under argon. ^{1}H NMR and ^{13}C NMR spectra were recorded with a Bruker Avance II 250 MHz or an Avance III HD Nanobay 400 MHz spectrometer in $CDCl_3$, MeOD or DMSO-*d6*. The chemical shifts are reported in parts per million (δ scale), and all coupling constant (J) values are in Hertz (Hz). The following abbreviations were used to denote the multiplicities: s (singlet), d (doublet), t (triplet), q (quartet), m (multiplet), and dd (double doublet). IR absorption spectra were obtained with a Perkin–Elmer PARAGON 1000 PC instrument, and values are reported in cm^{-1}. HRMS were recorded with a Bruker maXis Q-Tof mass spectrometer. Optical rotations were recorded on a Jasco P2000 polarimeter at 20 °C. Monitoring of the reactions was performed with silica gel TLC plates with a fluorescent indicator. Spots were visualized with UV light at 254 nm and 356 nm. Column chromatography was performed with silica gel 60 (0.063–0.200 mm, Merck). Supercritical fluid chromatography (SFC) was employed to determine enantiomeric excesses of compounds **15**, **16** and **17**. Samples were dissolved in methanol at 1 mg mL-1. SFC analyses were carried out on an ACQUITY UPC instrument from Waters. The stationary phase was a Chiralpak IB-N5 or Chiralpak IE columns from Chiral Technologies (150 × 4.6 mm, 5 µm). Oven temperature was set at 25 °C, backpressure at 150 bar and flow rate at 2 mL min^{-1}. Data are reported as follows: column type, eluent, flow rate, temperature, backpressure, wavelength and retention times (tR). Automated flow reactions were performed using a Uniqsis FlowSyn (inner diameter ID: 1mm).

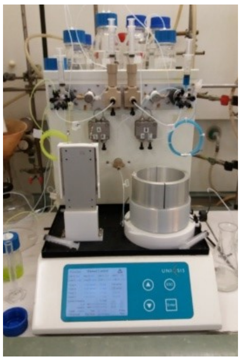

2- **Preparation of starting materials** (Compounds not in the text are represented with Roman numbers)

Esters **1**, **3**, **4** and **10** were prepared according to the literature.[1, 2]

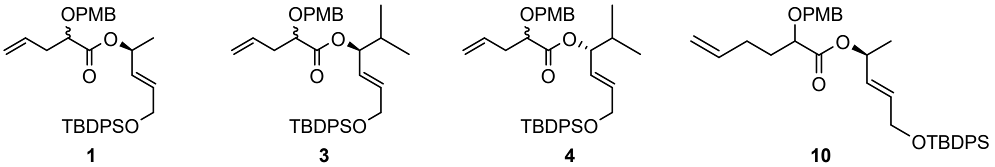

1 3 4 10

Chiral alcohols **I** and **II** as well as 2-(4-methoxybenzyloxy)-pent-4-enoic acid **III** were prepared as previously reported[2-4]

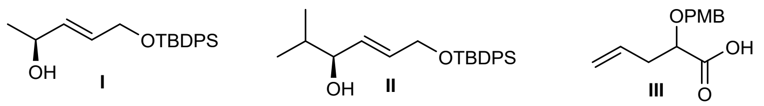

2-[(4-methoxyphenyl)methoxy]hex-5-enoic acid IV

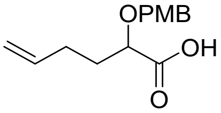

IV

This acid **IV** was prepared in a 5-step sequence starting from commercial glycidol. Protection of the alcohol was performed as reported by Sommer and co-workers[5] to give the THP derivative **V**. Opening of the epoxide was performed in the presence of allyl magnesium bromide and copper as reported by Donohue in 2008.[6] Protection of the alcohol as a PMB ether gave **VII** followed by removal of the THP protecting group (**VIII**). The desired acid **IV** was then prepared using an oxidation procedure reported by Shelke on the same substrate.[7]

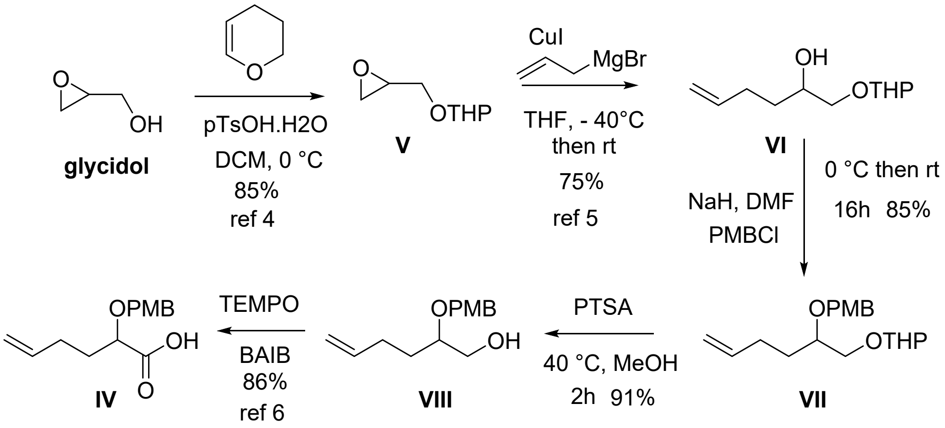

2-[2-[(4-methoxyphenyl)methoxy]hex-5-enoxy]tetrahydropyran VII

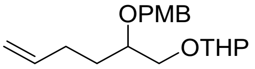

VII

Alcohol **VI** (1.80 g, 8.96 mmol) was suspended in 45 mL of dry DMF under Ar. The solution was cooled to 0 °C, and NaH (60% dispersion in oil, 0.540 g, 13.4 mmol, 1.5 equiv.) was added portion wise. The reaction was stirred for 30 at this temperature before addition of 4-methoxybenzyl chloride (1.69 g, 10.8 mmol, 1.45 mL, 1.2 equiv.) and a catalytic amount of tetrabutylammonium iodide (0.331 g, 0.89 mmol, 0.1 equiv.). The mixture was gradually warmed to rt and stirred overnight (16h). The reaction was carefully quenched with the addition of H_2O and taken up in EtOAc (100 mL). The organic layer was washed with water (3x) and a saturated NaCl solution before being dried over $MgSO_4$, filtered and concentrated. The crude reaction mixture was purified by silica gel column chromatography (PE/EtOAc 90/10) to give compound **VII** (2.45 g, 7.65 mmol, 85%) as a colorless oil (mixture of diastereoisomers). ^{1}H NMR (400 MHz, $CDCl_3$) δ 7.28 (d, *J* = 8.3 Hz, 2H), 6.87 (d, *J* = 8.5 Hz, 2H), 5.88 – 5.73 (m, 1H), 5.05 – 4.91 (m, 2H), 4.68 – 4.60 (m, 2H), 4.50 (dd, *J* = 11.3, 4.1 Hz, 1H), 3.90 – 3.72 (m, 5H), 3.64 – 3.55 (m, 1H), 3.55 – 3.42 (m, 2H), 2.27 – 2.05 (m, 2H), 1.90 – 1.78 (m, 1H), 1.76 – 1.47 (m, 7H). The ^{13}C spectra

showed two peaks for certain carbons. ^{13}C NMR (101 MHz, $CDCl_3$) δ (159.3, 159.3), (138.7, 138.7), (131.2, 131.2), (129.6, 129.5), (114.8, 114.8), 113.9, (99.3, 98.9), (77.3, 77.1), (72.0, 71.7), (70.5, 69.7), (62.3, 62.0), 55.4, (31.5, 31.5), (30.8, 30.7), (29.9, 29.8), 25.6, (19.6, 19.4). IR (ATR diamond, cm^{-1}) ν: 3127, 3006, 2856, 2223, 1637, 1546, 1446, 1336, 1238, 779, 692, 606. HRMS (EI-MS) m/z calcd for $C_{19}H_{28}O_4$ $[M+Na]^+$: 343.1879, found: 343.1885.

2-[(4-methoxyphenyl)methoxy]hex-5-en-1-ol VIII

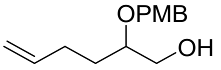

VIII

The protected diol **VII** (2.2 g, 6.80 mmol) was dissolved in MeOH (50 mL). *p*-Toluenesulfonic acid (0.863 g, 3.40 mmol, 0.5 equiv.) was added and the reaction mixture was heated to 40 °C until disappearance of the starting material by tlc (2-3 h). EtOAc (150 mL) and a saturated $NaHCO_3$ solution (50 mL) were added and the aqueous layer was extracted with EtOAc. The combined organic layers were washed with a saturated NaCl solution before being dried over $MgSO_4$, filtered and concentrated. The crude reaction mixture was purified by silica gel column chromatography (PE/EtOAc 80/20) to give alcohol **VIII** (1.45 g, 6.14 mmol, 90%) as a colorless oil. This product was identical to the one described by Shelke and co-workers.[7]

2-[(4-Methoxyphenyl)methoxy]hexanoic acid IX

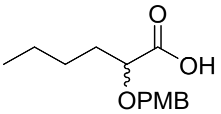

IX

This product was obtained in a three-step procedure without intermediate purification starting from the commercial available racemic 2-hydroxy hexanoic acid. The acid (0.385 g, 2.91 mmol) was dissolved in 5 mL of dry MeOH. Four drops of sulfuric acid were then added and the mixture was stirred for 4h. The solution was evaporated to dryness and EtOAc (15 mL) was added. The organic layer was extracted three times with saturated NaCl solution. The resulting solution was dried over $MgSO_4$, filtered and evaporated under reduced pressure to afford the desired hydroxy methyl ester in quantitative yield. The hydroxy methyl ester and PMB-trichloroacetimidate (1.63 g, 5.77 mmol, 1.20 mL, 2.0 equiv.) were dissolved in dry DCM (6 mL) under Ar. Camphor-10-sulphonic acid (0.067 g, 0.058 mmol, 0.1 equiv.) was then added and the resulting mixture was stirred overnight at room temperature. A mixture of 100 mL of EP/AcOEt (80/20) was added and the suspension was filtered over celite. The filtrate was evaporated under reduced pressure and the resulting oil was taken on to the next step without further purification. The crude PMB ester was then diluted in THF (8.0 mL) and cooled to 0 °C. A solution of LiOH (8.0 mL of a 1M aqueous solution) was added to the reaction and the mixture was stirred for 1 h. The solvent was partially evaporated, and the aqueous layer extracted with dichloromethane. The aqueous layer was then acidified with 1M HCl until pH 1. The desired acid was extracted with EtOAc, dried over $MgSO_4$ and concentrated. The desired acid **IX** (0.551 g, 75% 3 steps) was obtained as a colorless oil. ^1H NMR (400 MHz, $CDCl_3$) δ 9.81 (s, 1H), 7.29 (d, J = 8.5 Hz, 2H), 6.89 (d, J = 8.1 Hz, 2H), 4.66 (d, J = 11.2 Hz, 1H), 4.42 (d, J = 11.2 Hz, 1H), 3.97 (t, J = 6.2 Hz, 1H), 3.81 (s, 3H), 1.80 (q, J = 7.1 Hz, 2H), 1.47 – 1.36 (m, 2H), 1.35 – 1.24 (m, 2H), 0.89 (t, J = 7.2 Hz, 3H). ^{13}C NMR (101 MHz, $CDCl_3$) δ 178.2, 159.6, 129.9 (2 x CH), 129.3, 114.0 (2 x CH), 77.4, 72.3, 55.4, 32.5, 27.3, 22.4, 14.0. IR (ATR diamond, cm^{-1}) ν: 2957, 1716, 1682, 1604, 1513, 1001, 1247, 1095, 1027, 908, 843, 729, 633. HRMS (EI-MS) m/z calcd for $C_{14}H_{20}NaO_4$ $[M+Na]^+$: 275.1254, found: 275.1256.

2-[(4-methoxyphenyl)methoxy]-3-phenyl-propanoic acid X

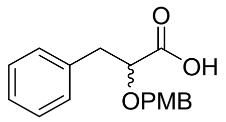 **X**

This acid was made in a three-step procedure without intermediate purification. The commercial racemic 3-phenyl lactic acid (1.5 g, 9.03 mmol) was dissolved in 8 mL of dry MeOH. Four drops of sulfuric acid were then added and the mixture was stirred for 4h. The solution was evaporated to dryness and EtOAc (20 mL) was added. The organic layer was extracted three times with saturated NaCl solution. The resulting solution was dried over $MgSO_4$, filtered and evaporated under reduced pressure to afford the desired hydroxy methyl ester in quantitative yield. The ester was used without further purification in the next step. The hydroxy methyl ester and PMB-trichloroacetimidate (5.10 g, 18 mmol, 3.75 mL, 2.0 equiv.) were dissolved in dry DCM under Ar. Camphor-10-sulphonic acid (0.210 g, 0.903 mmol, 0.1 equiv.) was then added and the resulting mixture was stirred overnight at room temperature. A mixture of 100 mL of EP/AcOEt (80/20) was added and the suspension was filtered over celite. The filtrate was evaporated under reduced pressure to give the crude product. The ester was diluted in THF (22 mL) and cooled to 0 °C. A solution of LiOH (22 mL of a 1M aqueous solution) was added to the reaction and the mixture was stirred for 1 h. The solvent was partially evaporated, and the aqueous layer extracted with dichloromethane. The aqueous layer was then acidified with 1M HCl until pH 1. The desired acid was extracted with EtOAc, dried over $MgSO_4$ and concentrated. The desired acid **X** (1.58 g, 5.50 mmol, 61%, 3 steps) was obtained as a colorless oil. ^1H NMR (400 MHz, $CDCl_3$) δ 7.34 – 7.20 (m, 5H), 7.08 (d, *J* = 8.3 Hz, 2H), 6.81 (d, *J* = 8.3 Hz, 2H), 4.56 (d, *J* = 11.3 Hz, 1H), 4.36 (d, *J* = 11.3 Hz, 1H), 4.17 (dd, *J* = 8.6, 4.3 Hz, 1H), 3.79 (s, 3H), 3.15 (dd, *J* = 14.1, 4.1 Hz, 1H), 3.02 (dd, *J* = 14.1, 8.4 Hz, 1H). ^{13}C NMR (101 MHz, $CDCl_3$) δ 176.0, 159.7, 136.8, 129.8 (2 x CH), 129.7 (2 x CH), 128.8, 128.5 (2 x CH), 127.0, 114.0 (2 x CH), 78.4, 72.8, 55.4, 39.0. IR (ATR diamond, cm^{-1}) ν: 3004, 2933, 1756, 1729, 1608, 1510, 1245, 1173, 1080, 1033, 1015, 1004, 838, 821, 720, 700, 647, 637. HRMS (EI-MS) m/z calcd for $C_{17}H_{18}NaO_4$ $[M+Na]^+$: 309.1097, found: 309.1095.

3- General procedures

General procedure A: DCC esterification

The chiral alcohol (1 equiv.) and the desired carboxylic acid (1.1 equiv.) were dissolved in dry methylene chloride (0.07 M) with a catalytic amount of DMAP (0.1 equiv.). The flask was cooled to 0 °C and stirred for 10 minutes. A solution of DCC (1.2 equiv.) in CH_2Cl_2 was slowly added dropwise. The mixture was allowed to warm to rt and was stirred overnight. The solvent was evaporated, a minimal amount of EtOAc was added, and the reaction was filtered on Celite. The crude product was purified by silica gel column chromatography to afford the desired ester.

General procedure B for the Claisen-Ireland rearrangement in batch mode:

n-BuLi (3 equiv., 2.3 M solution in hexanes) was added to a solution of HMDS (4 equiv.) in dry toluene (0.3 M) cooled to 0 °C under argon and stirred for 15 min. The solution was then further cooled to -78 °C. The starting ester (1 equiv.) dissolved in dry toluene (0.01 M) was added dropwise via a canula to the reaction vessel at –78 °C. The reaction was stirred for 45 min before freshly distilled TMSCl (3 equiv.) was introduced via syringe and maintained at this temperature for an extra 10 min before gradually warming to rt. After 2 h, the reaction was quenched by a saturated aqueous solution of ammonium chloride. The aqueous layer was extracted with diethyl ether, dried over $MgSO_4$, filtered and concentrated. The crude acid was immediately treated with a solution of freshly prepared

diazomethane in ether[8] to give the corresponding methyl ester which was purified by silica gel column chromatography.

General procedure C for the Claisen-Ireland rearrangement (3 pumps protocol) in continuous flow:

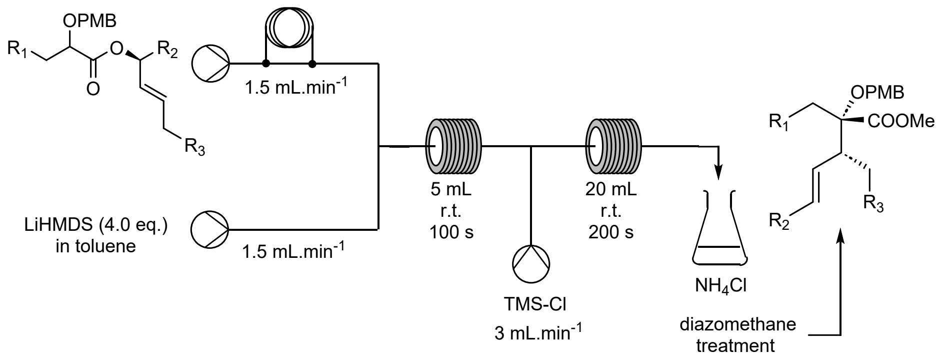

A solution A of ester derivative (1.0 equiv; C = 0.36 mol·L^{-1} in toluene) was pumped at 1.5 mL·min^{-1} and mixed using a standard T-piece with a second solution B containing LiHMDS (4.0 equiv, C = 0.72 mol·L^{-1} in toluene) also pumped at 1.5 mL·min^{-1}. The combined solution was then passed through a 5 mL linear reactor with a residence time of 100 seconds at room temperature. The entire output from the reactor was mixed using a second standard T-piece with a third solution C containing TMSCl (2.5 equiv, 0.45 mol/L in toluene , 3.0 mL.min^{-1}) pumped at 3.0 mL·min^{-1}. The solution was then passed through a 20 mL linear reactor with a residence time of 200 seconds at room temperature. The solution was collected in a quench solution of sat. aq. NH_4Cl and the organic phase was extracted with ethyl acetate. The combined organic layers were washed with a solution of a 1M HCl, dried over $MgSO_4$, filtered and concentrated in vacuo. The resulting crude acid was then treated with a freshly prepared solution of diazomethane in ether (10 equiv.) [8] to give the corresponding methyl ester which was purified by silica gel column chromatography.

[(E,1R)-4-[tert-butyl(diphenyl)silyl]oxy-1-isopropyl-but-2-enyl] 2-[(4-methoxyphenyl) methoxy] hex-5-enoate 5

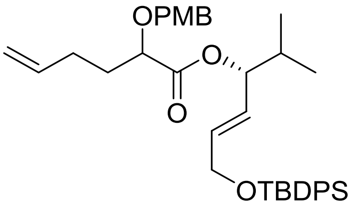

5

The general esterification procedure A was used with 2-[(4-methoxyphenyl)methoxy]hex-5-enoic acid **IV** (0.193 g, 0.77 mmol, 1.1 equiv.) and alcohol **I** (0.258 g, 0.70 mmol). Purification by silica gel column chromatography (100/0 to 90/10 PE/EtOAc) gave the desired ester **5** (0.201 g, 48%) as a colorless oil. ^{1}H NMR (400 MHz, $CDCl_3$) δ 7.70 – 7.62 (m, 4H), 7.45 – 7.33 (m, 6H), 7.30 – 7.24 (m, 2H), 6.89 – 6.82 (m, 2H), 5.85 – 5.69 (m, 3H), 5.20 (t, *J* = 5.6 Hz, 1H), 5.03 – 4.93 (m, 2H), 4.66 (d, *J* = 11.2 Hz, 1H), 4.31 (d, *J* = 11.1 Hz, 1H), 4.22 (s, 2H), 3.93 (t, *J* = 6.5 Hz, 1H), 3.78 (s, 3H), 2.29 – 2.10 (m, 2H), 1.97 – 1.80 (m, 3H), 1.06 (d, *J* = 1.5 Hz, 9H), 0.97 – 0.89 (m, 6H). ^{13}C NMR (101 MHz, $CDCl_3$) δ 172.3, 159.5, 137.7, 135.6 (4 x CH), 134.9, 133.7 (2 x C), 133.1, 129.9, 129.9, 129.8, 127.8 (5 x CH), 126.1, 115.5, 113.9 (2 x CH), 79.4, 77.3, 72.0, 63.7, 55.4, 32.4, 32.3, 29.6, 26.9 (3 x CH_3), 19.4, 18.3 (2 x CH_3). IR (ATR diamond, cm^{-1}) ν: 2960, 2932, 2857, 1746, 1725, 1613, 1514, 1249, 1096, 1035, 987, 929, 820. HRMS (EI-MS) m/z calcd for $C_{37}H_{48}NaO_5Si$ [M+Na]$^+$: 623.3163, found: 623.3164.

[(E,1R)-4-[tert-butyl(diphenyl)silyl]oxy-1-isopropyl-but-2-enyl] 2-[(4-methoxyphenyl)methoxy] hexanoate 6

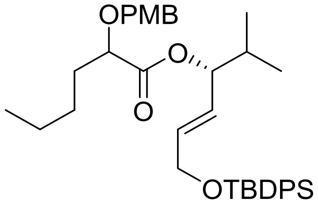

6

The general esterification procedure A was used with 2-[(4-methoxyphenyl)methoxy]hexanoic acid **IX** (0.287 g, 1.14 mmol) and alcohol **II** (0.461 g, 1.25 mmol, 1.1 equiv.). Purification by silica gel column chromatography (95/5 PE/EtOAc) gave the desired ester **6** (0.301 g, 44%) as a colorless oil (mixture of diastereoisomers). ^{1}H NMR (400 MHz, $CDCl_3$) δ 7.73 – 7.63 (m, 4H), 7.44 – 7.32 (m, 6H), 7.27 (d, *J* = 8.1 Hz, 2H), 6.85 (d, *J* = 7.9 Hz, 2H), 5.88 – 5.73 (m, 2H), 5.23 – 5.16 (m, 1H), 4.64 (t, *J* = 11.2 Hz, 1H), 4.32 (d, *J* = 11.2 Hz, 1H), 4.24 – 4.19 (m, 2H), 3.90 (t, *J* = 6.1 Hz, 1H), 3.78 (s, 3H), 1.98 – 1.86 (m, 1H), 1.80 – 1.69 (m, 2H), 1.49 – 1.19 (m, 4H), 1.06/1.05 (2 x s, 9H), 0.93 (t, *J* = 6.3 Hz, 6H), 0.90 – 0.83 (m, 3H). The ^{13}C spectra showed two peaks for certain carbons. ^{13}C NMR (101 MHz, $CDCl_3$) δ (172.6, 172.5), (159.5, 159.4), (135.6, 135.6), 134.9, (133.7, 133.7), 133.4, 133.0, (130.0, 130.0), (129.8, 129.8), 127.8, (126.1, 126.0), (113.9, 113.9), (79.4, 79.3), (78.1, 78.0), (71.9, 71.9), (63.6, 63.6), 55.4, (32.9, 32.9), (32.3, 32.3), (27.6, 27.5), (26.9, 26.9), 22.5, 19.4, (18.3, 18.3), 14.0. IR (ATR diamond, cm^{-1}) ν: 2953, 2932, 2858, 1746, 1613, 1514, 1247, 1095, 1036, 987, 820. HRMS (EI-MS) m/z calcd for $C_{37}H_{50}NaO_5Si$ $[M+Na]^+$: 625.3320, found: 625.3323.

[(1R)-1-vinylhexyl] 2-[(4-methoxyphenyl)methoxy] hexanoate 7

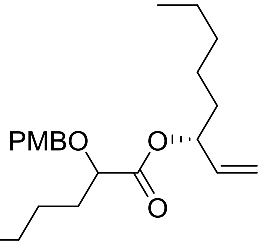

7

The general esterification procedure A was used with 2-[(4-methoxyphenyl)methoxy]hexanoic acid **IX** (0.217 g, 0.86 mmol) and commercially available (R)-(-)-1-octen-3-ol (0.121 g, 0.95 mmol, 1.1 equiv.). Purification by silica gel column chromatography (95/5 PE/EtOAc) gave the desired ester **7** (0.285 g, 91%) as a colorless oil (mixture of diastereoisomers). ^{1}H NMR (400 MHz, $CDCl_3$) δ 7.27 (d, *J* = 8.0 Hz, 2H), 6.87 (d, *J* = 8.0 Hz, 2H), 5.86 – 5.73 (m, 1H), 5.37 – 5.22 (m, 2H), 5.18 (d, *J* = 10.5 Hz, 1H), 4.63 (dd, *J* = 11.2, 4.6 Hz, 1H), 4.33 (d, *J* = 11.3 Hz, 1H), 3.92 – 3.85 (m, 1H), 3.80 (s, 3H), 1.80 – 1.54 (m, 4H), 1.48 – 1.22 (m, 10H), 0.87 (t, *J* = 6.7 Hz, 6H). ^{13}C NMR (101 MHz, $CDCl_3$) δ 172.5, 159.5, (136.6, 136.5), (129.9, 129.9), 129.8, 129.8, 117.3, 116.9, 113.9, (78.0, 78.0), (75.4, 75.3), (71.9, 71.9), 55.4, 34.3, (32.9, 32.8), 31.6, 27.5, (24.9, 24.9), (22.7, 22.6), 22.5, (14.1, 14.1), 14.0. IR (ATR diamond, cm^{-1}) ν: 2954, 2931, 2860, 1746, 1729, 1612, 1513, 1245, 1120, 1096, 1035, 988, 821. HRMS (EI-MS) m/z calcd for $C_{22}H_{34}NaO_4$ $[M+Na]^+$: 385.2349, found: 385.2351.

[1R)-1-vinylhexyl] 2-[(4-methoxyphenyl)methoxy]hex-5-enoate 8

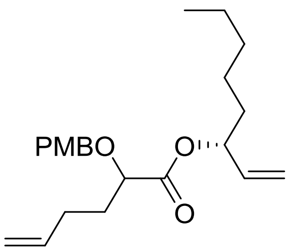

8

The general esterification procedure A was used with acid **IV** (0.253 g, 1.01 mmol) and (R)-(-)-1-octen-3-ol (0.143 g, 1.1 mmol, 1.1 equiv.). Purification by silica gel column chromatography (97/3 PE/EtOAc) gave the desired ester **8** (0.259 g, 71%) as a colorless oil (mixture of diastereoisomers). ^{1}H NMR (400 MHz, $CDCl_3$) δ 7.27 (d, *J* = 8.2 Hz, 2H), 6.87 (d, *J* = 8.0 Hz, 2H), 5.85 – 5.70 (m, 2H), 5.38 – 5.31 (m, 1H), 5.30 – 5.14 (m, 2H), 5.04 – 4.92 (m, 2H), 4.64 (d, *J* = 11.1 Hz, 1H), 4.32 (d, *J* = 11.2 Hz, 1H), 3.96 – 3.88 (m, 1H), 3.80 (s, 3H), 2.26 – 2.09 (m, 2H), 1.89 – 1.76 (m, 2H), 1.71 – 1.53 (m, 2H), 1.40 – 1.22 (m, 6H), 0.94 – 0.84 (m, 3H). ^{13}C NMR (101 MHz, $CDCl_3$) δ 172.3, 159.5, 137.6, 136.5, 136.5, 129.8 (2 x CH), 117.0, 115.5, 113.9 (2 x CH), 77.2, 75.4, 72.0, 55.4, 34.3, 32.4, 31.6, 29.6, 24.9, 22.6, 14.1. IR (ATR diamond, cm^{-1}) ν: 2958, 2931, 2860, 1745, 1726, 1613, 1513, 11247, 1173, 1104, 1035, 989, 914, 821. HRMS (EI-MS) m/z calcd for $C_{22}H_{32}NaO_4$ $[M+Na]^+$: 383.2193, found: 383.2194.

[(1R)-1-vinylhexyl] 2-[(4-methoxyphenyl)methoxy]-3-phenyl-propanoate 9

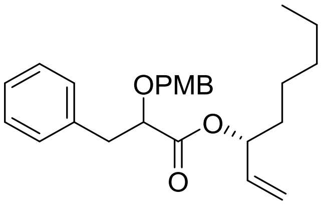

9

The general esterification procedure A was used with acid **X** (0.400 g, 1.40 mmol) and (R)-(-)-1-octen-3-ol (0.206 g, 1.6 mmol, 1.1 equiv.). Purification by silica gel column chromatography (95/5 PE/EtOAc) gave the desired ester **9** (0.329 g, 59%) as a colorless oil (mixture of diastereoisomers). ^{1}H NMR (400 MHz, $CDCl_3$) δ 7.30 – 7.18 (m, 5H), 7.07 (d, *J* = 7.1 Hz, 2H), 6.79 (d, *J* = 7.4 Hz, 2H), 5.84 – 5.64 (m, 1H), 5.34 – 5.27 (m, 1H), 5.27 – 5.08 (m, 2H), 4.59 (d, *J* = 11.5 Hz, 1H), 4.29 (d, *J* = 11.4 Hz, 1H), 4.16 – 4.06 (m, 1H), 3.79 (s, 3H), 3.13 – 2.97 (m, 2H), 1.68 – 1.48 (m, 2H), 1.35 – 1.19 (m, 6H), 0.93 – 0.84 (m, 3H). The ^{13}C spectra showed two peaks for certain carbons. ^{13}C NMR (101 MHz, $CDCl_3$) δ (171.8, 171.7), 159.4, 137.3 (2 x C), (136.5, 136.3), (129.7, 129.7, 2 x CH), 129.6 (2 x CH), (128.4, 128.4, 2 x CH), (126.7, 126.7), (117.4, 117.2), 113.8 (2 x CH), (79.1, 79.1), (75.7, 75.7), (72.1, 72.1), 55.4, (39.5, 39.4), (34.3, 34.2), 31.6, (24.8, 24.8), (22.7, 22.6), 14.1. IR (ATR diamond, cm^{-1}) ν: 2931, 2859, 1745, 1612, 1513, 1247, 1172, 1101, 1034, 978, 820, 745. HRMS (EI-MS) m/z calcd for $C_{25}H_{32}NaO_4$ $[M+Na]^+$: 419.2193, found: 419.2194.

Rearrangement products:

Methyl (E,2S,3S)-2-allyl-3-[[tert-butyl(diphenyl)silyl]oxymethyl]-2-[(4-methoxyphenyl) methoxy] hex-4-enoate 2

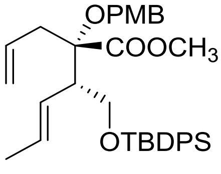

2

General procedure C was used with ester **1** (0.170 g, 0.31 mmol) and 2.0 equiv. of freshly prepared LiHMDS. After treatment with diazomethane, the crude methyl ester was purified by silica gel column chromatography (95/5 PE/EtOAc) to give the desired rearrangement product **2** (109 mg, 62%) as an

86/14 mixture of diastereoisomers. ^{1}H NMR (400 MHz, $CDCl_3$) δ 7.67 – 7.61 (m, 4H), 7.43 – 7.29 (m, 6H), 7.23 (d, *J* = 8.1 Hz, 2H), 6.84 (d, *J* = 8.0 Hz, 2H), 5.91 – 5.75 (m, 1H), 5.63 – 5.49 (m, 1H), 5.35 (dd, *J* = 15.3, 9.6 Hz, 1H), 5.15 – 4.97 (m, 2H), 4.62 – 4.35 (m, 2H), 4.08 (dd, *J* = 10.3, 4.0 Hz, 0.14H), 3.86 – 3.74 (m, 3.85H), 3.69 – 3.55 (m, 4H), 2.92 – 2.81 (m, 1H), 2.79 – 2.54 (m, 2H), 1.69 (d, *J* = 6.3 Hz, 3H), 1.01 (s, 9H). The ^{13}C spectra showed two peaks for certain carbons. ^{13}C NMR (101 MHz, $CDCl_3$) δ (173.5, 172.9), (159.0, 158.9), (135.9, 135.8), 135.8, (134.1, 134.0), 134.0, 133.8, (131.5, 131.0), (130.0, 129.8), (129.6, 129.5), (128.8, 128.7), 128.4, 127.8, 127.7, 127.7, (118.6, 118.1), 113.7, (83.2, 83.1), (66.8, 66.2), (64.0, 63.2), 55.4, 53.4, (51.7, 51.6), 39.8, 27.0, 19.4, 18.3. IR (ATR diamond, cm^{-1}) ν: 2931, 2856, 1738, 1613, 1514, 1427, 1247, 1110, 1036, 822. HRMS (EI-MS) m/z calcd for $C_{35}H_{44}O_5Si$ $[M+H]^+$: 573.3031, found: 573.3026.

Methyl (E,2S,3S)-2-allyl-3-[[tert-butyl(diphenyl)silyl]oxymethyl]-2-[(4-methoxyphenyl)methoxy]-6-methyl-hept-4-enoate 11

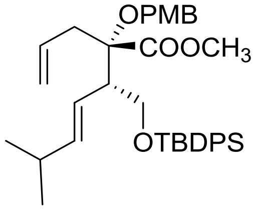

11

General procedure C was used with ester **3** (0.174 g, 0.30 mmol). After treatment with diazomethane, the crude methyl ester was purified by silica gel column chromatography (95/5 PE/EtOAc) to give the desired rearrangement product **11** (156 mg, 62%) as a single diastereoisomer (dr > 99%). ^{1}H and ^{13}C spectra were identical to those reported in the literature using a batch synthesis.[2]

Methyl (E,2R,3S)-2-allyl-3-[[tert-butyl(diphenyl)silyl]oxymethyl]-2-[(4-methoxyphenyl)methoxy]-6-methyl-hept-4-enoate 12

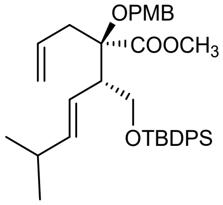

12

General procedure C was used with ester **4** (0.190 g, 0.32 mmol). After treatment with diazomethane, the crude methyl ester was purified by silica gel column chromatography (95/5 PE/EtOAc) to give the desired rearrangement product **12** (129 mg, 66%) as a single diastereoisomer (dr > 99%). ^{1}H and ^{13}C spectra were identical to those reported in the literature using a batch synthesis.[2]

Methyl (E,2R,3S)-2-but-3-enyl-3-[[tert-butyl(diphenyl)silyl]oxymethyl]-2-[(4-methoxyphenyl) methoxy]-6-methyl-hept-4-enoate 13

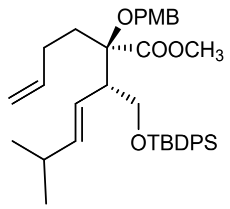

13

General procedure C was used with ester **5** (0.100 g, 0.17 mmol). After treatment with diazomethane, the crude methyl ester was purified by silica gel column chromatography (90/10 up to 85/15 PE/EtOAc) to give the desired rearrangement product **13** (53 mg, 52%) as a single diastereoisomer (dr > 99%). ^{1}H NMR (400 MHz, $CDCl_3$) δ 7.65 (d, *J* = 6.9 Hz, 4H), 7.43 – 7.29 (m, 6H), 7.23 (d, *J* = 7.1 Hz, 2H), 6.83 (d, *J*

= 7.6 Hz, 2H), 5.79 – 5.66 (m, 1H), 5.56 (dd, J = 15.3, 6.6 Hz, 1H), 5.44 – 5.26 (m, 1H), 5.04 – 4.85 (m, 2H), 4.51 (s, 2H), 3.84 – 3.75 (m, 4H), 3.67 (t, J = 9.5 Hz, 1H), 3.59 (s, 3H), 2.92 – 2.79 (m, 1H), 2.37 – 2.26 (m, 1H), 2.21 – 2.09 (m, 1H), 2.08 – 1.97 (m, 1H), 1.97 – 1.78 (m, 2H), 1.07 – 0.95 (m, 15H). ^{13}C NMR (101 MHz, $CDCl_3$) δ 173.8, 159.0, 142.4, 138.4, 135.8 (4 x CH), 134.0, 134.0, 131.5, 129.6, 129.6, 128.7 (2 x CH), 127.7 (4 x CH), 123.9, 114.6, 113.7 (2 x CH), 83.2, 66.1, 64.1, 55.4, 52.3, 51.6, 33.8, 31.6, 28.3, 27.0 (3 x CH_3), 22.8, 22.7, 19.4. IR (ATR diamond, cm^{-1}) ν: 2956, 2931, 2858, 1737, 1514, 1246, 1035, 911, 820. $[\alpha]^{20}_D = -10.7$ (c 1.0M, $CHCl_3$). HRMS (EI-MS) m/z calcd for $C_{38}H_{51}O_5Si$ $[M+H]^+$: 615.3500, found: 615.3502.

Methyl (E,2R,3S)-2-butyl-3-[[tert-butyl(diphenyl)silyl]oxymethyl]-2-[(4-methoxyphenyl)methoxy]-6-methyl-hept-4-enoate 14

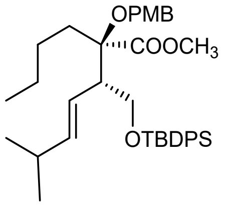

14

General procedure C was used with ester **6** (0.100 g, 0.17 mmol). After treatment with diazomethane, the crude methyl ester was purified by silica gel column chromatography (90/10 PE/EtOAc) to give the desired rearrangement product **14** (59 mg, 58%) as a 90:10 mixture of diastereoisomers. ^1H NMR (400 MHz, $CDCl_3$) δ 7.70 – 7.59 (m, 4H), 7.44 – 7.28 (m, 6H), 7.24 (d, J = 7.9 Hz, 2H), 6.83 (d, J = 7.9 Hz, 2H), 5.55 (dd, J = 15.4, 6.8 Hz, 1H), 5.34 (dd, J = 15.5, 9.3 Hz, 1H), 4.56 – 4.45 (m, 1.85H), 4.56 – 4.29 (m, 0.20H), 3.79 (s, 3H), 3.74 – 3.62 (m, 2H), 3.58 (s, 3H), 2.89 – 2.80 (m, 1H), 2.38 – 2.24 (m, 1H), 1.87 – 1.68 (m, 2H), 1.29 – 1.18 (m, 4H), 1.05 – 0.96 (m, 15H), 0.85 (t, J = 6.8 Hz, 3H). ^{13}C NMR (101 MHz, $CDCl_3$) δ 174.1, 158.9, 142.2, 135.8 (4 x CH), 134.1, 134.0, 131.7, 129.6, 129.6, 128.6 (2 x CH), 127.7 (4 x CH), 124.1, 113.7 (2 x CH), 83.5, 66.0, 64.1, 55.4, 52.5, 51.5, 34.3, 31.6, 27.0 (3 x CH_3), 26.1, 23.3, 22.8, 22.7, 19.4, 14.1. IR (ATR diamond, cm^{-1}) ν: 2955, 2929, 2858, 1737, 1613, 1513, 1247, 1110, 1037, 821. HRMS (EI-MS) m/z calcd for $C_{38}H_{53}O_5Si$ $[M+H]^+$: 617.3657, found: 617.3662.

Methyl (E,2R)-2-butyl-2-[(4-methoxyphenyl)methoxy]dec-4-enoate 15

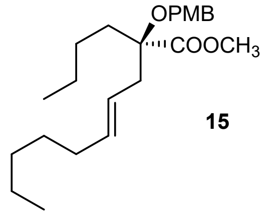

15

General procedure C was used with ester **7** (0.140 g, 0.39 mmol). After treatment with diazomethane, the crude methyl ester was purified by silica gel column chromatography (90/10 to 80/20 PE/EtOAc) to give the desired rearrangement product **15** (120 mg, 83%) as a mixture of enantiomers (er 97:7). ^1H NMR (400 MHz, $CDCl_3$) δ 7.31 (d, J = 8.2 Hz, 2H), 6.87 (d, J = 7.7 Hz, 2H), 5.57 – 5.47 (m, 1H), 5.42 – 5.32 (m, 1H), 4.42 – 4.31 (m, 2H), 3.79 (s, 3H), 3.73 (s, 3H), 2.64 – 2.49 (m, 2H), 1.99 (q, J = 7.0 Hz, 2H), 1.81 (t, J = 7.7 Hz, 2H), 1.39 – 1.19 (m, 10H), 0.92 – 0.84 (m, 6H). ^{13}C NMR (101 MHz, $CDCl_3$) δ 174.4, 159.2, 134.9, 130.8, 129.3 (2 x CH), 123.7, 113.9 (2 x CH), 83.2, 66.0, 55.4, 51.9, 37.6, 34.4, 32.8, 31.5, 29.3, 25.3, 23.0, 22.7, 14.2, 14.1. IR (ATR diamond, cm^{-1}) ν: 2955, 2926, 2857, 1733, 1614, 1514, 1247, 1206, 1037, 971, 821. HRMS (EI-MS) m/z calcd for $C_{23}H_{37}O_4$ $[M+H]^+$: 377.2686, found: 377.2690. SFC analyze: Chiralpak IE , CO_2-MeOH 97:03 (v/v), 2 mL/min, 25°C , 150 bars, 254 nm and tr=5.16 min.

Methyl (E,2R)-2-but-3-enyl-2-[(4-methoxyphenyl)methoxy]dec-4-enoate 16

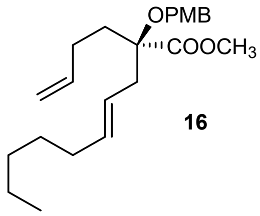

16

General procedure C was used with ester **8** (0.125 g, 0.35 mmol). After treatment with diazomethane, the crude methyl ester was purified by silica gel column chromatography (90/10 PE/EtOAc) to give the desired rearrangement product **16** (66 mg, 49%) as a mixture of enantiomers (er 94:6). ^{1}H NMR (400 MHz, $CDCl_3$) δ 7.31 (d, *J* = 8.0 Hz, 2H), 6.87 (d, *J* = 8.0 Hz, 2H), 5.86 – 5.73 (m, 1H), 5.58 – 5.48 (m, 1H), 5.43 – 5.31 (m, 1H), 5.06 – 4.91 (m, 2H), 4.40 (s, 2H), 3.80 (s, 3H), 3.74 (s, 3H), 2.66 – 2.51 (m, 2H), 2.21 – 2.08 (m, 1H), 2.07 – 1.87 (m, 5H), 1.39 – 1.20 (m, 6H), 0.88 (t, J = 6.4 Hz, 3H). ^{13}C NMR (101 MHz, $CDCl_3$) δ 174.2, 159.3, 138.1, 135.2, 130.7, 129.3 (2 x CH), 123.5, 114.8, 113.9 (2 x CH), 82.8, 66.1, 55.4, 51.9, 37.8, 33.8, 32.8, 31.5, 29.3, 27.5, 22.7, 14.2. IR (ATR diamond, cm^{-1}) ν: 2954, 2926, 2856, 1733, 1614, 1514, 1247, 1173, 1036, 972, 821. HRMS (EI-MS) m/z calcd for $C_{23}H_{35}O_4$ $[M+H]^+$: 375.2530, found: 375.2530. SFC analysis: Chiralpak IE, CO_2-MeOH 97:03 (v/v), 2 mL/min, 25 °C, 150 bars, 254 nm and tr=5.02 min.

Methyl (E,2S)-2-benzyl-2-[(4-methoxyphenyl)methoxy]dec-4-enoate 17

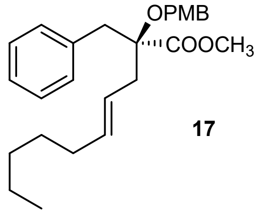

17

General procedure C was used with ester **9** (0.100 g, 0.25 mmol). After treatment with diazomethane, the crude methyl ester was purified by silica gel column chromatography (80/20 PE/EtOAc) to give the desired rearrangement product **17** (87 mg, 84%) as a single enantiomer (er > 99%). $[\alpha]^{20}_D$ = +18.3 (*c* 0.64, $CHCl_3$). ^{1}H NMR (400 MHz, $CDCl_3$) δ 7.26 – 7.06 (m, 7H), 6.80 (d, *J* = 7.9 Hz, 2H), 5.56 – 5.44 (m, 1H), 5.45 – 5.35 (m, 1H), 4.41 (s, 2H), 3.73 (s, 3H), 3.57 (s, 3H), 3.06 (s, 2H), 2.58 (dd, *J* = 15.1, 6.1 Hz, 1H), 2.46 (dd, *J* = 15.1, 7.3 Hz, 1H), 1.96 (q, *J* = 7.0 Hz, 2H), 1.33 – 1.16 (m, 6H), 0.81 (t, *J* = 6.5 Hz, 3H). ^{13}C NMR (101 MHz, $CDCl_3$) δ 173.6, 159.2, 136.3, 135.5, 130.6, 130.4 (2 x CH), 129.2 (2 x CH), 128.1 (2 x CH), 126.8, 123.4, 113.8 (2 x CH), 83.9, 66.4, 55.4, 51.8, 41.6, 36.6, 32.9, 31.5, 29.3, 22.7, 14.2. IR (ATR diamond, cm^{-1}) ν: 2925, 2854, 1736, 1614, 1514, 1247, 1110, 1034, 820, 700. HRMS (EI-MS) m/z calcd for $C_{26}H_{35}O_4$ $[M+H]^+$: 411.2530, found: 411.2532. SFC analyse : Chiralpak IB N5 , CO_2-MeOH 97:03 (v/v), 2 mL/min, 25°C , 150 bars, 254 nm and tr=8.97 min.

Large scale reaction

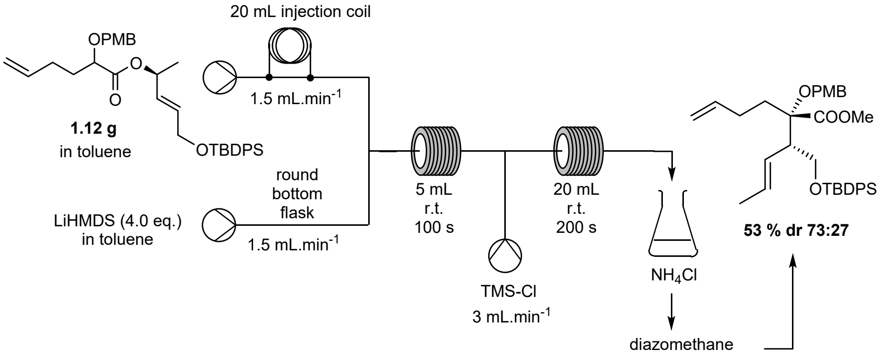

Methyl (E,2S,3S)-2-but-3-enyl-3-[[tert-butyl(diphenyl)silyl]oxymethyl]-2-[(4-methoxy-phenyl)methoxy]hex-4-enoate 18

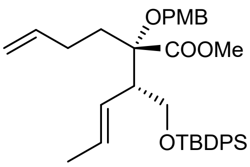

18

General procedure C was used with ester **10** (1.12 g, 1.95 mmol). After treatment with diazomethane, the crude methyl ester was purified by silica gel column chromatography (90/10 PE/EtOAc) to give the desired rearrangement product **18** (584 mg, 53%) as a 73:27 mixture of diastereoisomers. ^{1}H NMR (400 MHz, $CDCl_3$) δ 7.67 – 7.61 (m, 4H), 7.45 – 7.29 (m, 6H), 7.26 – 7.20 (m, 2H), 6.86 – 6.81 (m, 2H), 5.84 – 5.67 (m, 1H), 5.63 – 5.52 (m, 1H), 5.40 – 5.30 (m, 1H), 5.03 – 4.89 (m, 2H), 4.55 – 4.47 (m, 1.4H), 4.38 – 4.27 (m, 0.51H), 4.07 (dd, *J* = 10.0, 3.8 Hz, 0.24H), 3.80 (d, *J* = 2.3 Hz, 3.69H), 3.67 – 3.57 (m, 4H), 2.91 – 2.83 (m, 0.71H), 2.73 – 2.65 (m, 0.26H), 2.23 – 2.10 (m, 1H), 2.03 – 1.77 (m, 3H), 1.75 – 1.66 (m, 3H), 1.02 (s, 9H). The ^{13}C spectra showed two peaks for certain carbons and have been regrouped when possible. ^{13}C NMR (101 MHz, $CDCl_3$) δ (173.8, 173.2), (159.1, 159.0), (138.4, 138.3), (135.9, 135.8, 135.8, 4 x CH), (134.0, 133.9), (131.5, 131.0), 129.8, (129.6, 129.6), 129.6, 129.5, 128.8, 128.7, 128.5, 127.9, 127.8, 127.7, 127.7, 127.7, 127.6, 114.7, (113.8, 113.7), (83.1, 83.0), (66.1, 65.9), (64.1, 63.4), 55.4, (52.6, 51.8), 51.7, (34.1, 31.9), 28.4, 27.0, 27.0, 19.4, (18.4, 18.4). IR (ATR diamond, cm^{-1}) ν: 2931, 2856, 1737, 1613, 1514, 1427, 1247, 1111, 1036, 821. HRMS (EI-MS) m/z calcd for $C_{36}H_{46}NaO_5Si$ $[M+Na]^+$: 609.3007, found: 609.2999.

References

1. A. Français, O. Bedel, W. Picoul, A. Meddour, J. Courtieu and A. Haudrechy, *Tetrahedron: Asymmetry*, 2005, **16**, 1141-1155.
2. N. P. Probst, A. Haudrechy and K. Plé, *J. Org. Chem.*, 2008, **73**, 4338-4341.
3. P. Dorizon, G. Su, G. Ludvig, L. Nikitina, R. Paugam, J. Ollivier and J. Salaün, *J. Org. Chem.*, 1999, **64**, 4712-4724.
4. Y. Ichikawa, K. Tsuboi and M. Isobe, *J. Chem. Soc., Perkin Trans. 1*, 1994, DOI: 10.1039/p19940002791, 2791-2796.
5. S. Sommer, M. Kühn and H. Waldmann, *Adv. Synth. Catal.*, 2008, **350**, 1736-1750.
6. T. J. Donohoe, K. M. P. Wheelhouse , P. J. Lindsay-Scott, P. A. Glossop, I. A. Nash and J. S. Parker, *Angew. Chem. Int. Ed.*, 2008, **47**, 2872-2875.
7. A. M. Shelke and G. Suryavanshi, *Tetrahedron: Asymmetry*, 2016, **27**, 142-147.
8. P. Lombardi, *Chem. Ind.*, 1990, 708.

Parameter	Value
Solvent	CDCl3
Spectrometer Frequency	400.13

VII

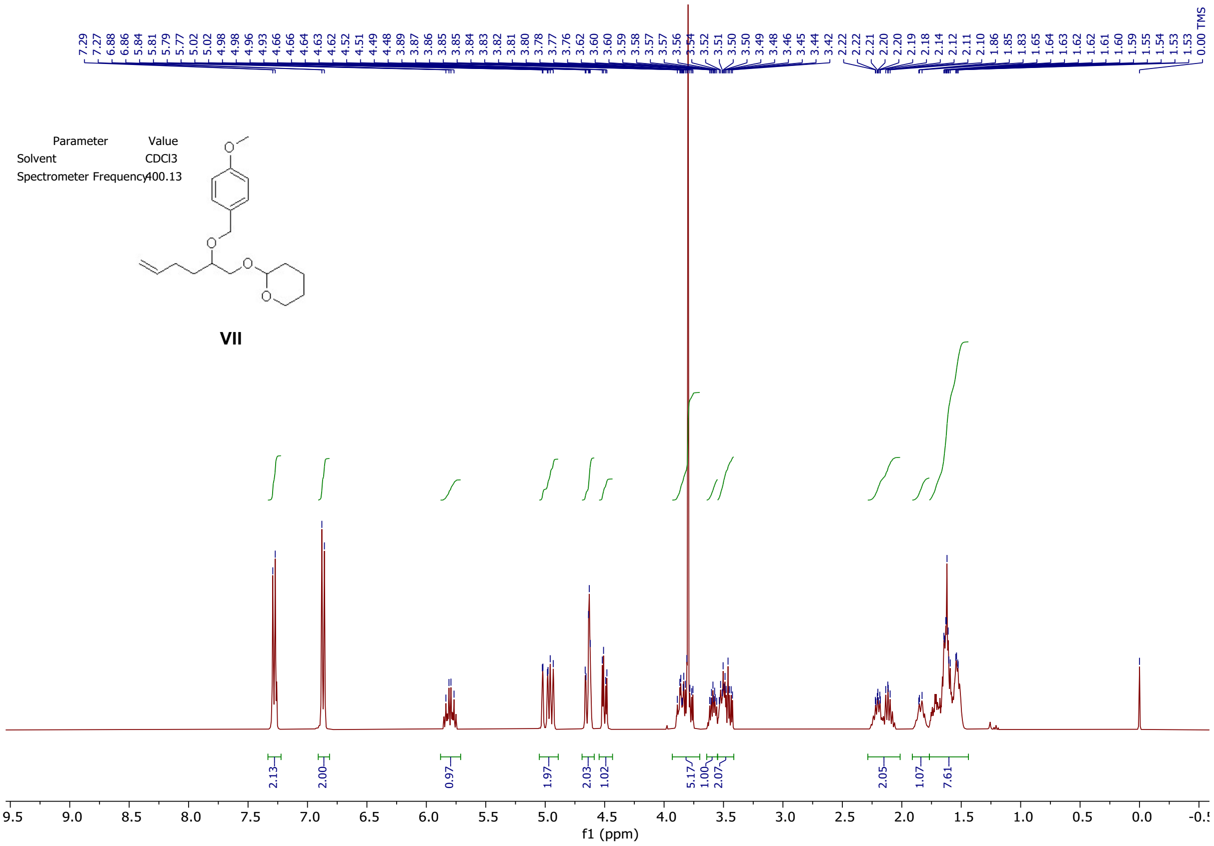

Parameter	Value
Solvent	CDCl3
Spectrometer Frequency	100.62

VII

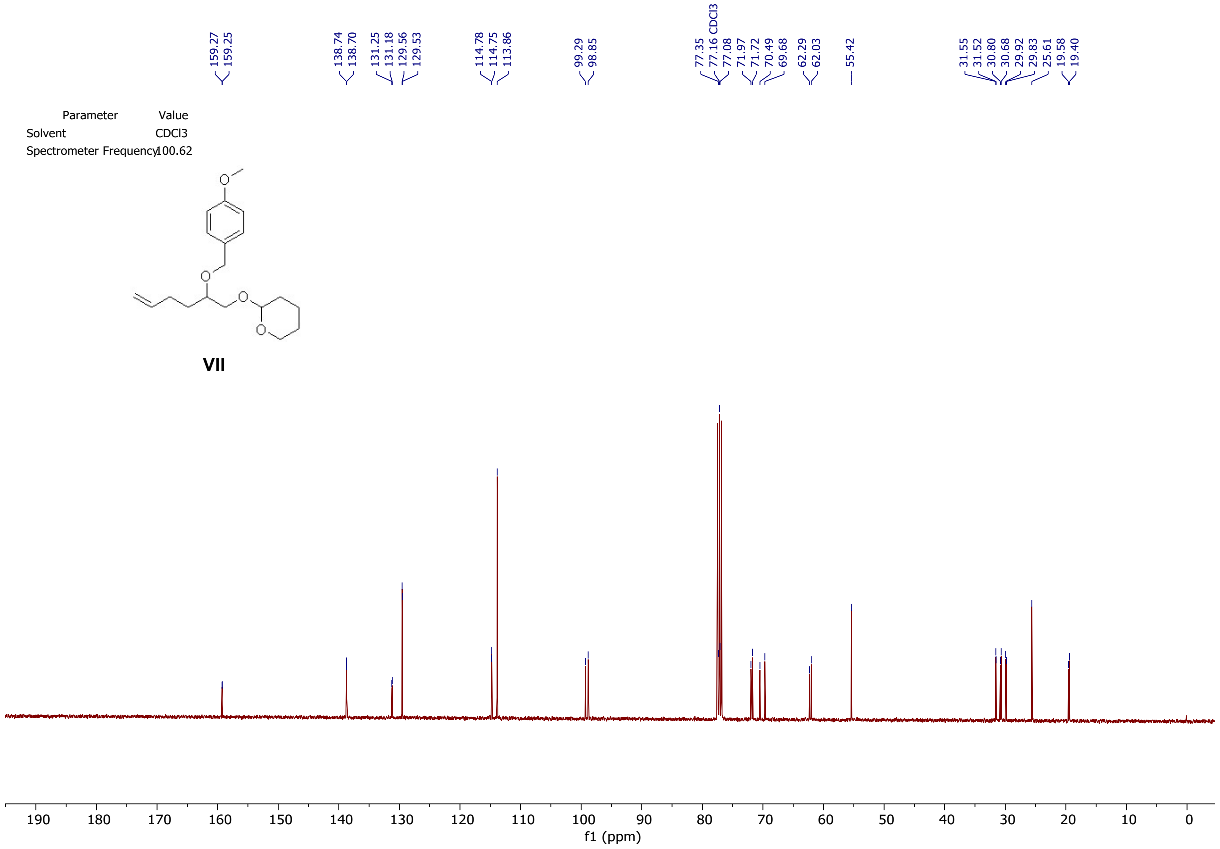

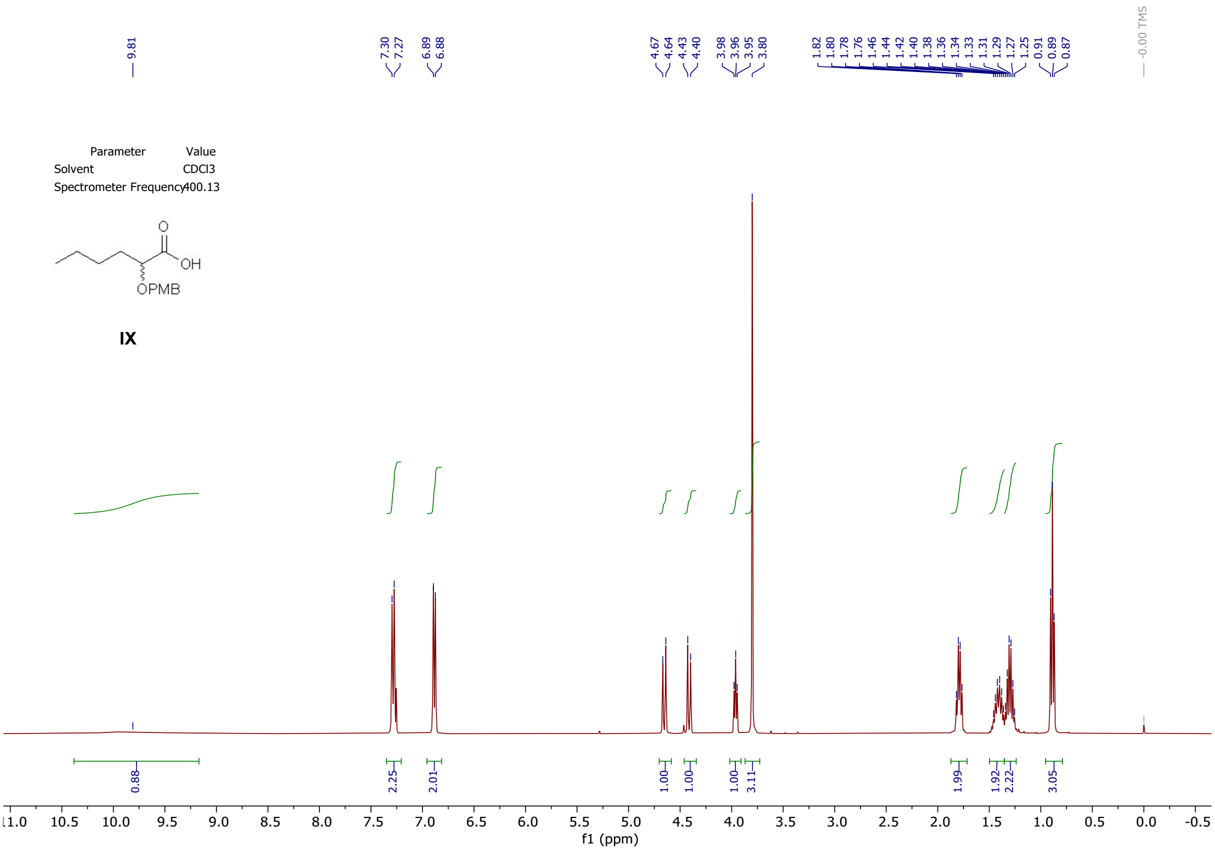
9.81
7.30
7.27
6.89
6.88
4.67
4.64
4.43
4.40
3.98
3.96
3.95
3.80
1.82
1.80
1.78
1.76
1.46
1.44
1.42
1.40
1.38
1.36
1.34
1.33
1.31
1.29
1.27
1.25
0.91
0.89
0.87
-0.00 TMS
Parameter Value
Solvent CDCl3
Spectrometer Frequency 400.13
OH
OPMB
IX
0.88
2.25
2.01
1.00
1.00
1.00
3.11
1.99
1.92
2.22
3.05
f1 (ppm)

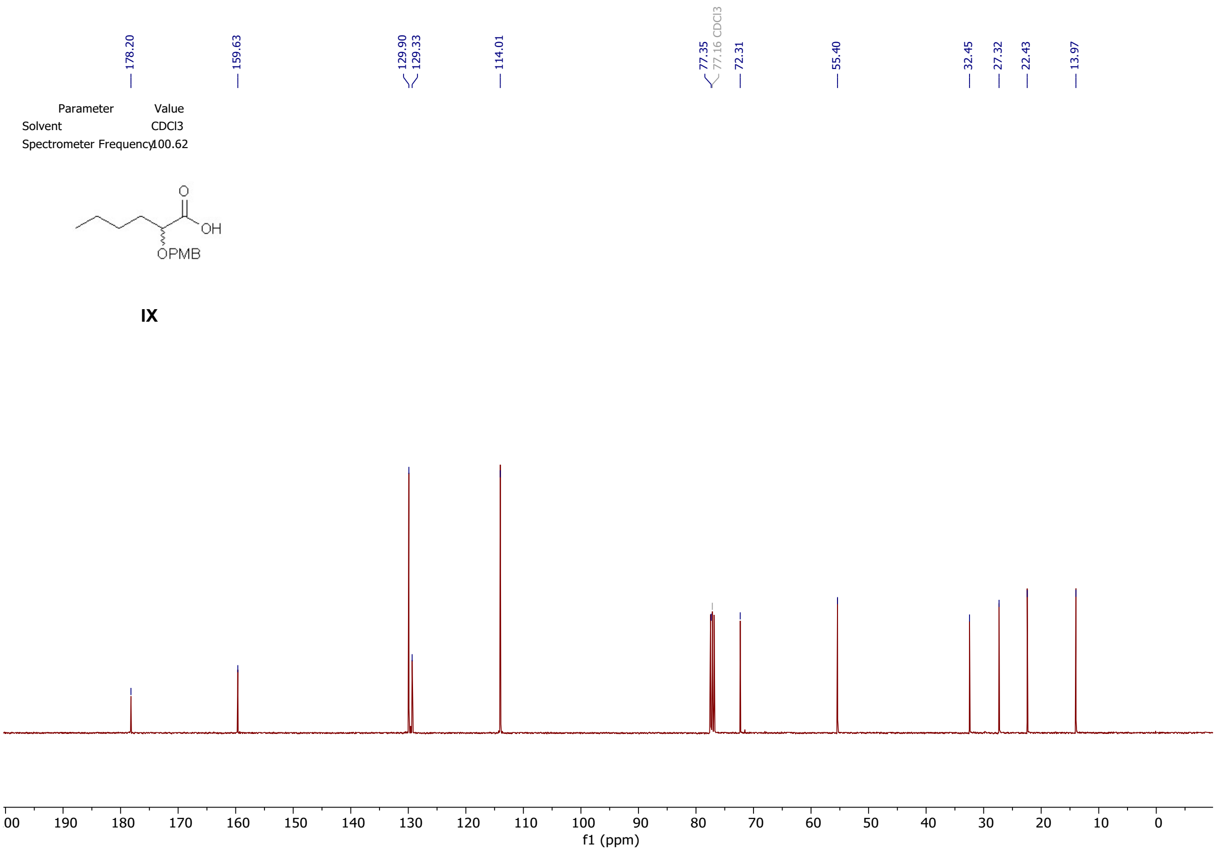
178.20
159.63
129.90
129.33
114.01
77.35
77.16 CDCl3
72.31
55.40
32.45
27.32
22.43
13.97
Parameter Value
Solvent CDCl3
Spectrometer Frequency 100.62
OH
OPMB
IX
f1 (ppm)

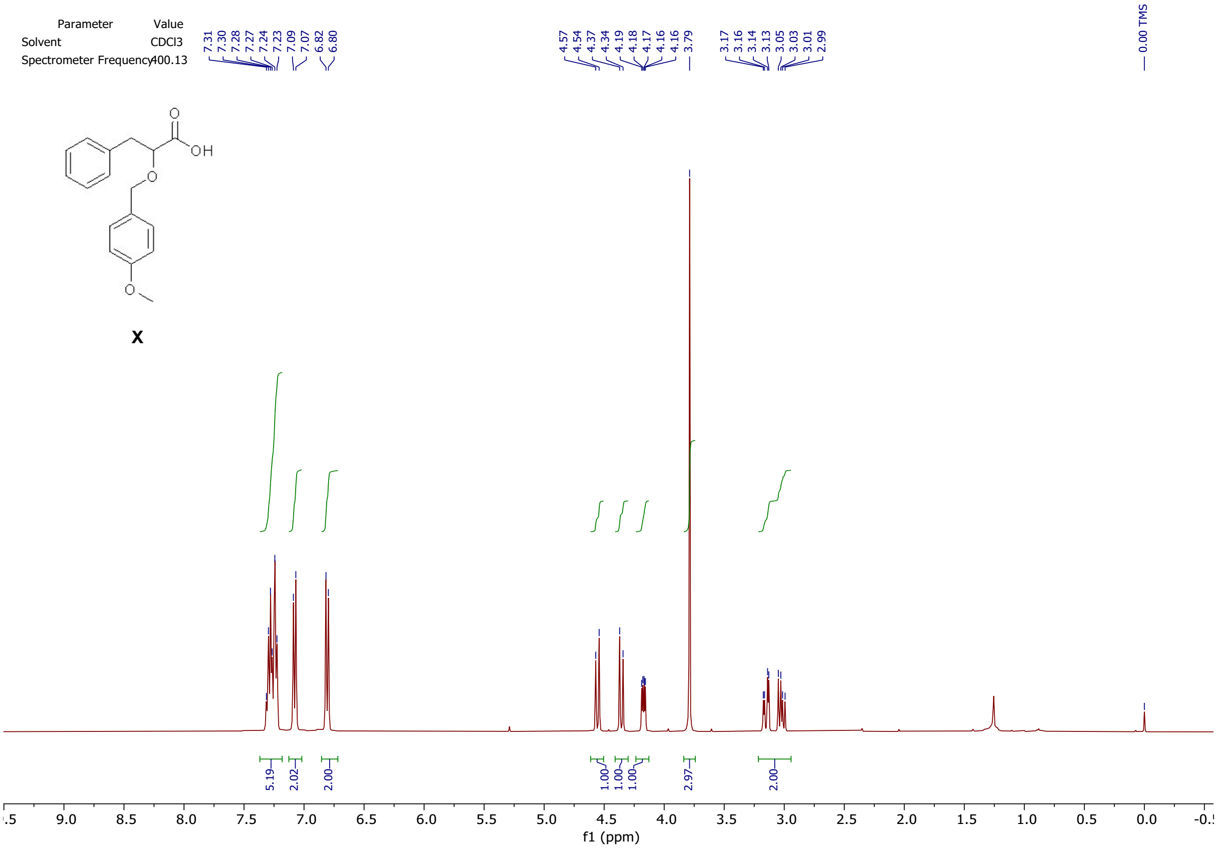
Parameter Value
Solvent CDCl3
Spectrometer Frequency 400.13
7.31
7.30
7.28
7.27
7.24
7.23
7.09
7.07
6.82
6.80
4.57
4.54
4.37
4.34
4.19
4.18
4.17
4.16
4.16
3.79
3.17
3.16
3.14
3.13
3.05
3.03
3.01
2.99
0.00 TMS
5.19
2.02
2.00
1.00
1.00
1.00
2.97
2.00
f1 (ppm)

X

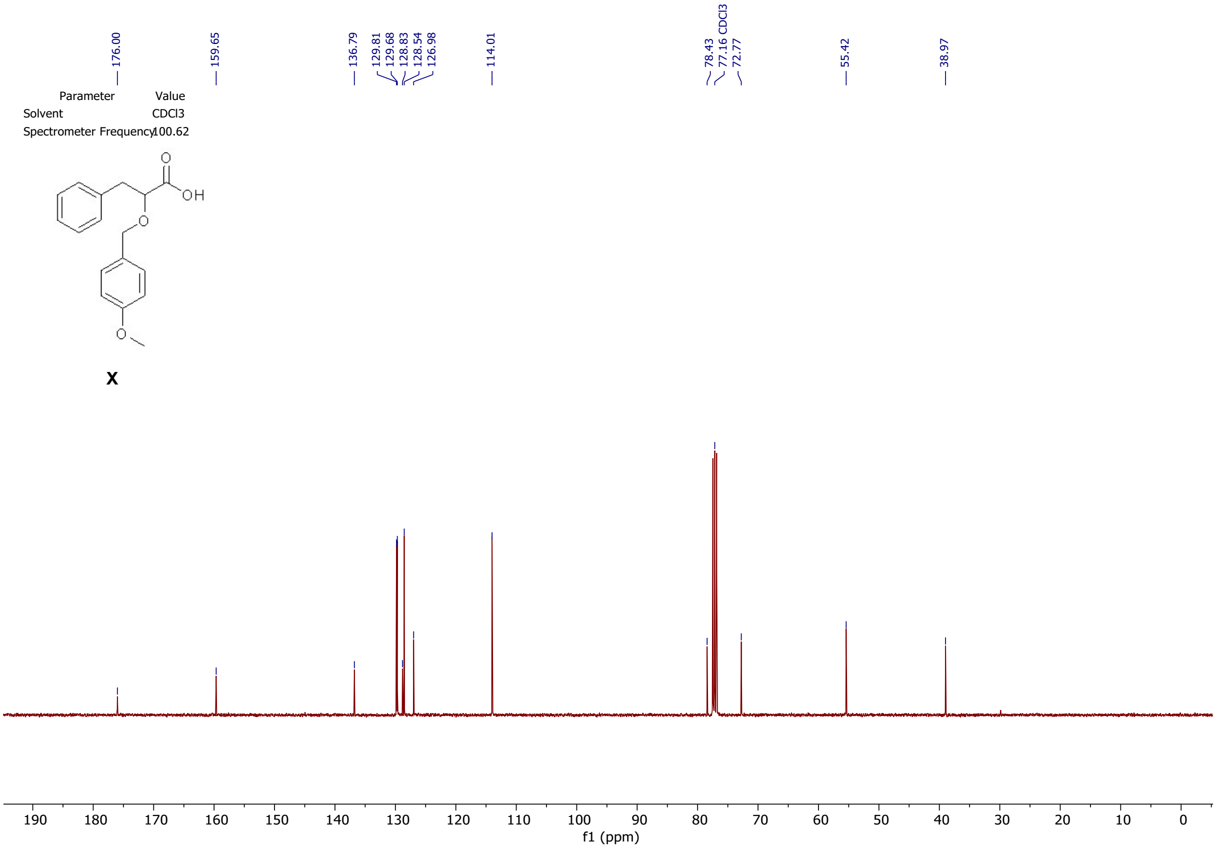
176.00
159.65
136.79
129.81
129.68
128.83
128.54
126.98
114.01
78.43
77.16 CDCl3
72.77
55.42
38.97
Parameter Value
Solvent CDCl3
Spectrometer Frequency 100.62
f1 (ppm)

X

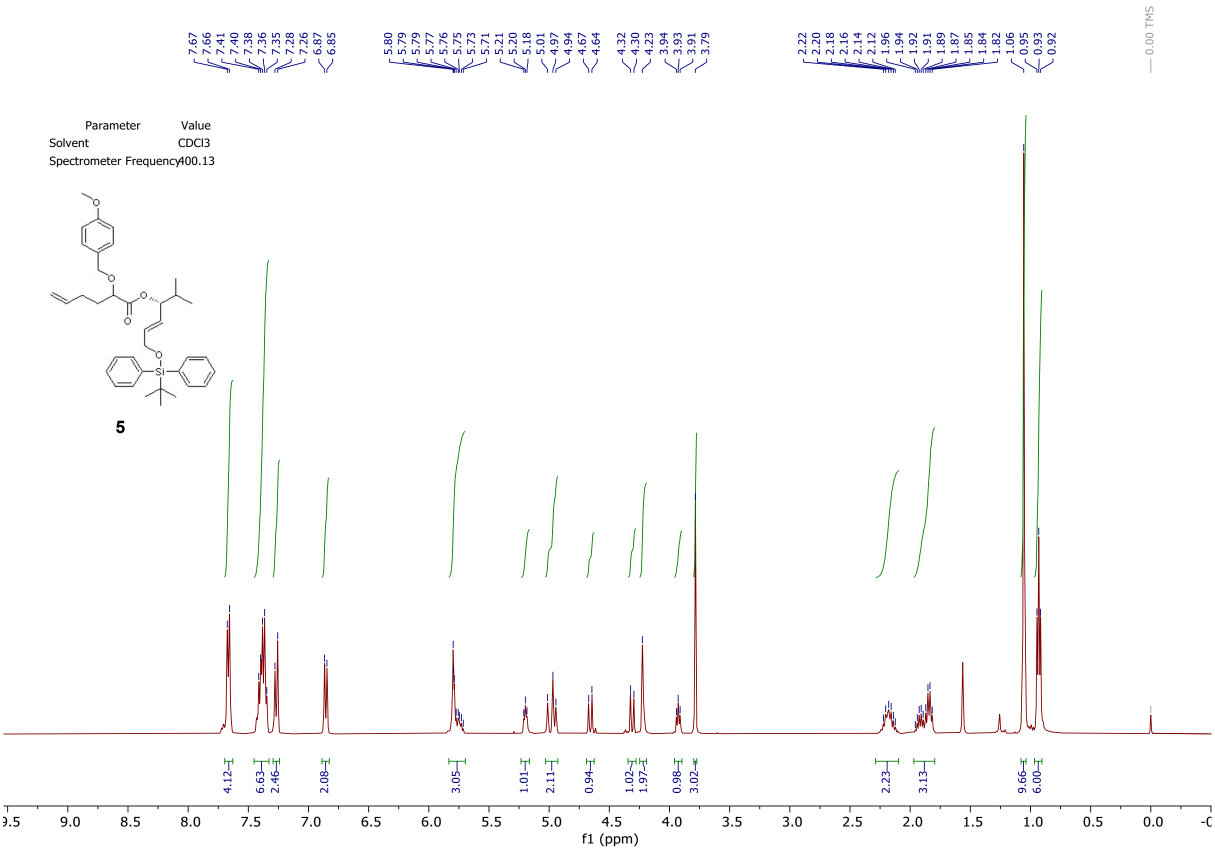

Parameter Value
Solvent CDCl3
Spectrometer Frequency 400.13
5
7.67 7.66 7.41 7.40 7.38 7.36 7.35 7.28 7.26 6.87 6.85
5.80 5.79 5.79 5.77 5.76 5.75 5.73 5.71 5.21 5.20 5.18 5.01 4.97 4.94 4.67 4.64 4.32 4.30 4.23 3.94 3.93 3.91 3.79
2.22 2.20 2.18 2.16 2.14 2.12 1.96 1.94 1.92 1.91 1.89 1.87 1.85 1.84 1.82 1.06 0.95 0.93 0.92
0.00 TMS
4.12 6.63 2.46 2.08 3.05 1.01 2.11 0.94 1.02 1.97 0.98 3.02 2.23 3.13 9.66 6.00
f1 (ppm)

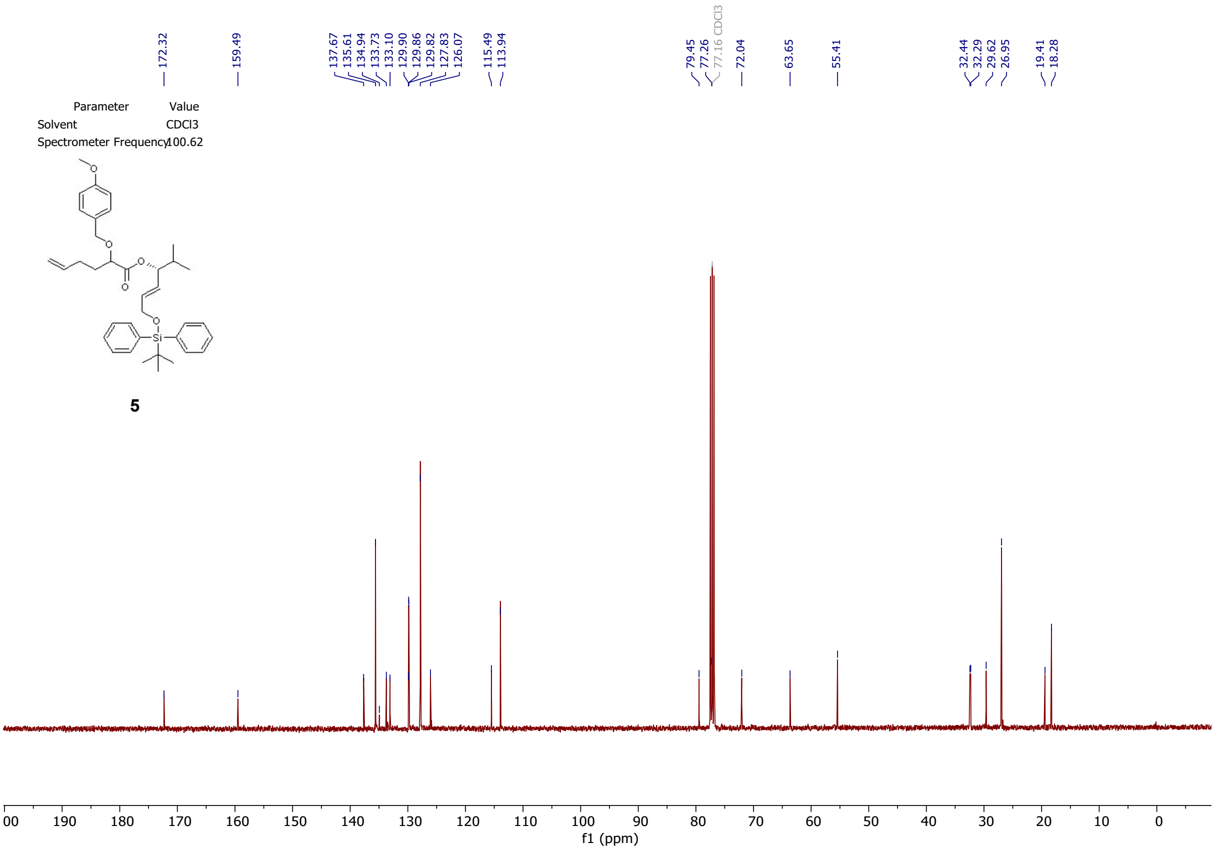

172.32
159.49
137.67 135.61 134.94 133.73 133.10 129.90 129.86 129.82 127.83 126.07
115.49 113.94
79.45 77.26 77.16 CDCl3
72.04
63.65
55.41
32.44 32.29 29.62 26.95
19.41 18.28
Parameter Value
Solvent CDCl3
Spectrometer Frequency 100.62
5
f1 (ppm)

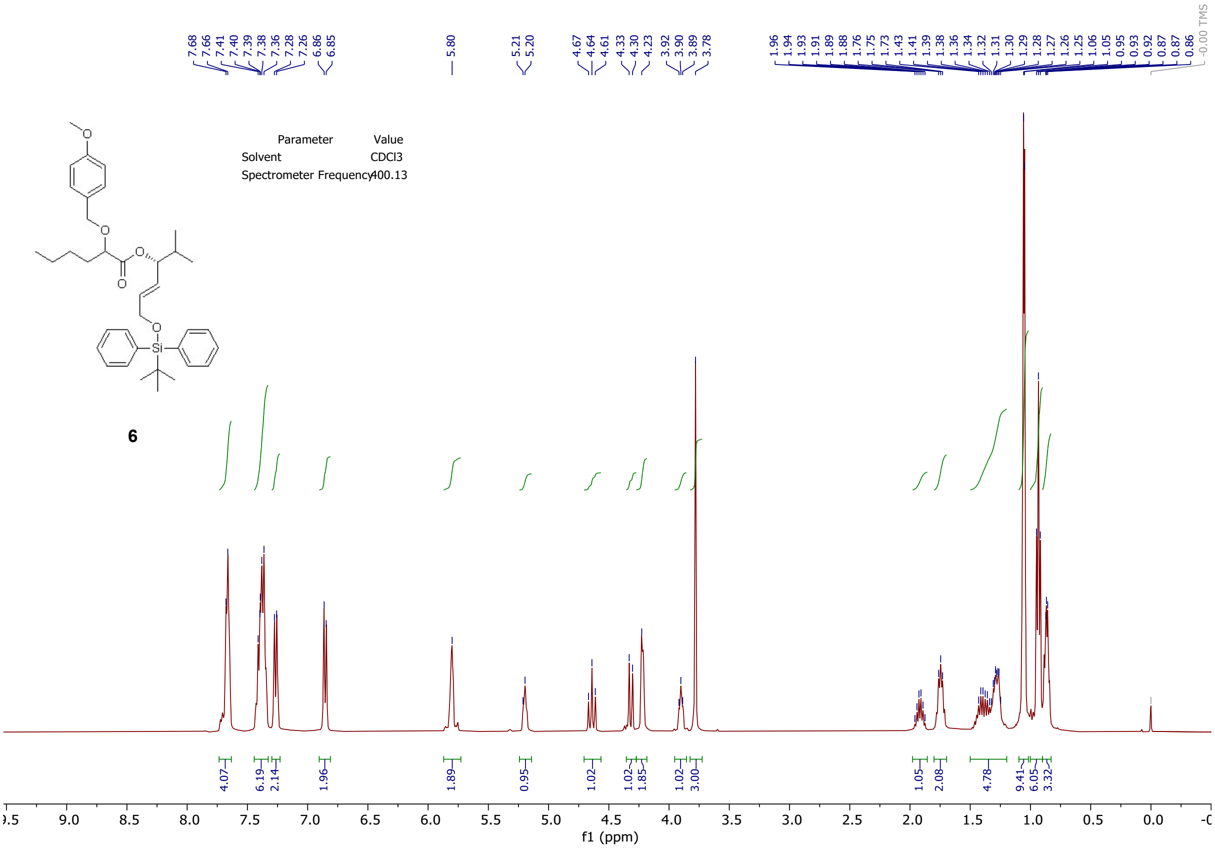
7.68 7.66 7.41 7.40 7.39 7.38 7.36 7.28 7.26 6.86 6.85
5.80
5.21 5.20
4.67 4.64 4.61 4.33 4.30 4.23 3.92 3.90 3.89 3.78
1.96 1.94 1.93 1.91 1.89 1.88 1.76 1.75 1.73 1.43 1.41 1.39 1.38 1.36 1.34 1.32 1.31 1.30 1.29 1.28 1.27 1.26 1.25 1.06 1.05 0.95 0.93 0.92 0.87 0.87 0.86
-0.00 TMS
Parameter Value
Solvent CDCl3
Spectrometer Frequency 400.13
O
O
O
O
O
Si
6
4.07 6.19 2.14 1.96 1.89 0.95 1.02 1.02 1.85 1.02 3.00 1.05 2.08 4.78 9.41 6.05 3.32
f1 (ppm)

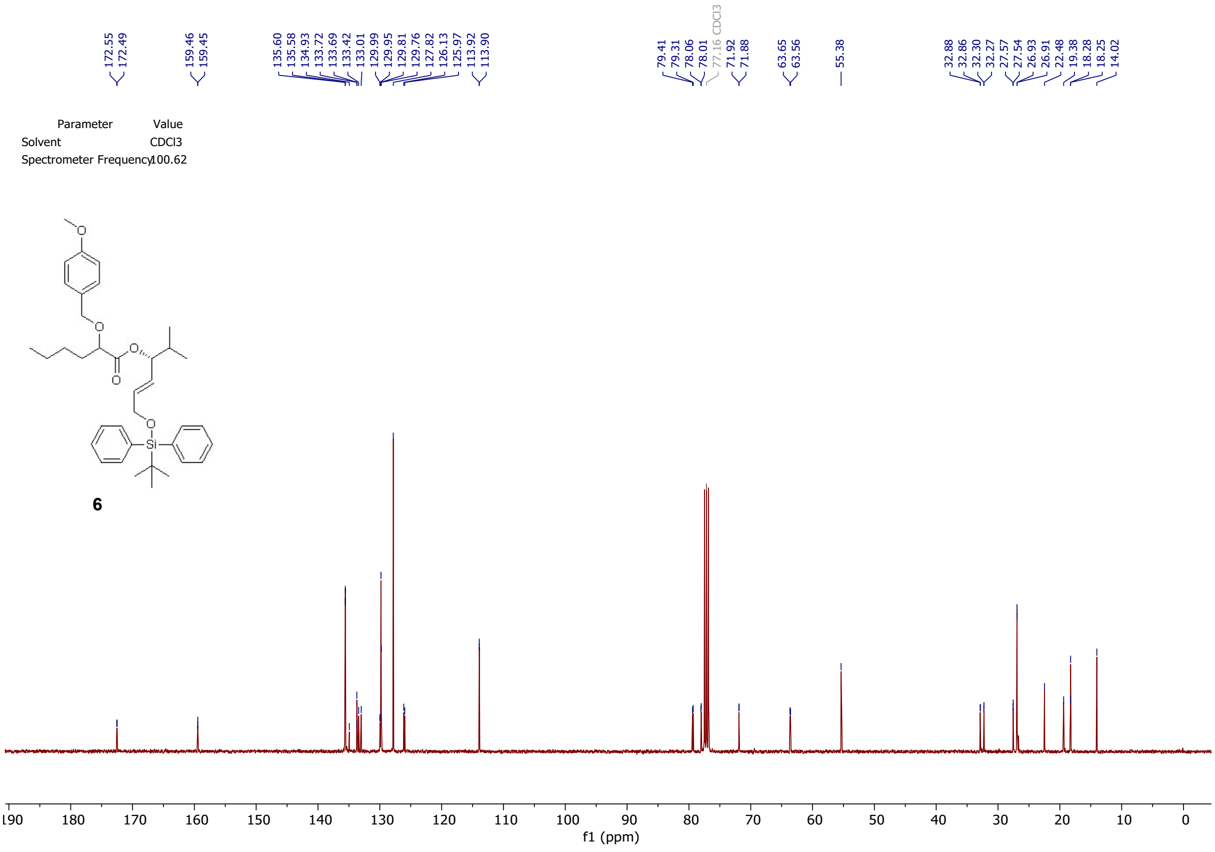
172.55 172.49
159.46 159.45
135.60 135.58 134.93 133.72 133.69 133.42 133.01 129.99 129.95 129.81 129.76 127.82 126.13 125.97 113.92 113.90
79.41 79.31 78.06 78.01 77.16 CDCl3 71.92 71.88
63.65 63.56
55.38
32.88 32.86 32.30 32.27 27.57 27.54 26.93 26.91 22.48 19.38 18.28 18.25 14.02
Parameter Value
Solvent CDCl3
Spectrometer Frequency 100.62
O
O
O
O
O
Si
6
f1 (ppm)

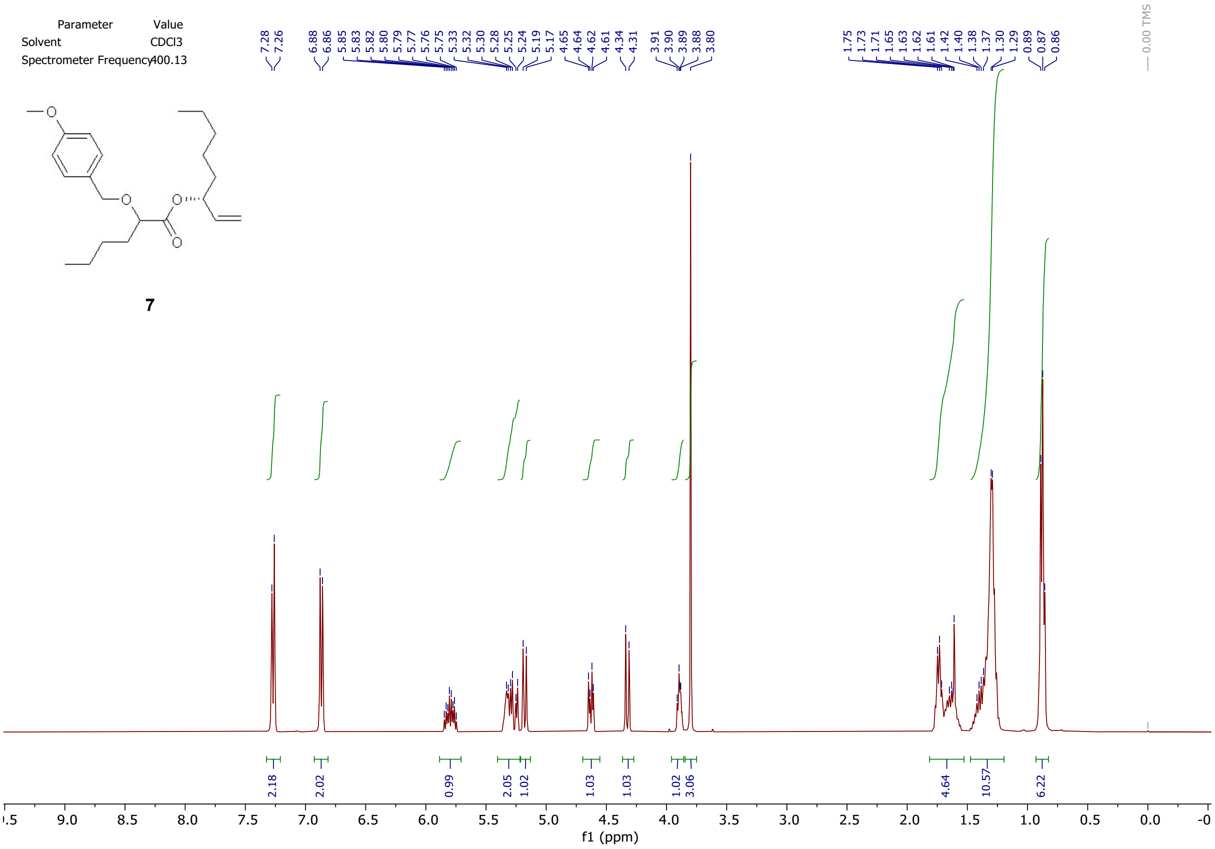
Parameter Value
Solvent CDCl3
Spectrometer Frequency 400.13
7
7.28 7.26 6.88 6.86 5.85 5.83 5.82 5.80 5.79 5.77 5.76 5.75 5.33 5.32 5.30 5.28 5.25 5.24 5.19 5.17 4.65 4.64 4.62 4.61 4.34 4.31 3.91 3.90 3.89 3.88 3.80
1.75 1.73 1.71 1.65 1.63 1.62 1.61 1.42 1.40 1.38 1.37 1.30 1.29 0.89 0.87 0.86
0.00 TMS
2.18 2.02 0.99 2.05 1.02 1.03 1.03 1.02 3.06 4.64 10.57 6.22
f1 (ppm)

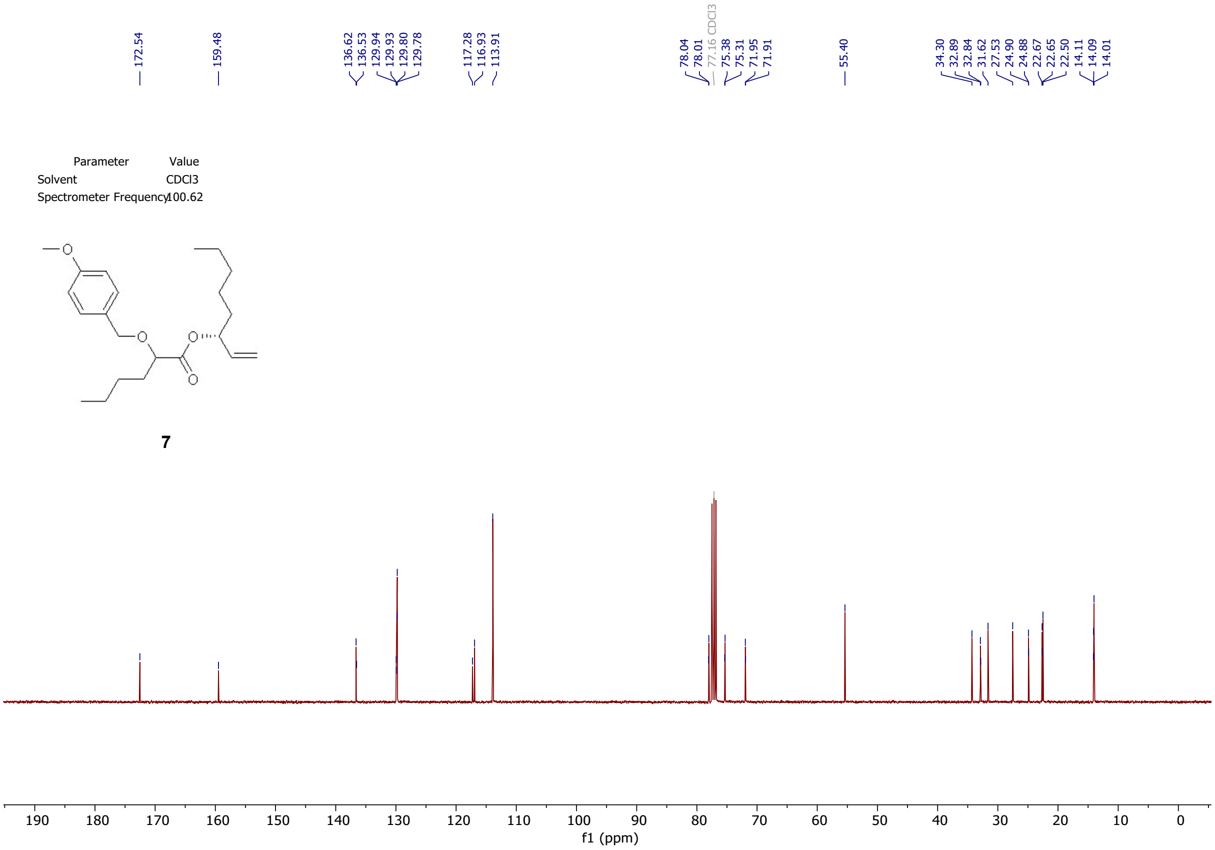
172.54 159.48 136.62 136.53 129.94 129.93 129.80 129.78 117.28 116.93 113.91
78.04 78.01 77.16 CDCl3 75.38 75.31 71.95 71.91 55.40
34.30 32.89 32.84 31.62 27.53 24.90 24.88 22.67 22.65 22.50 14.11 14.09 14.01
Parameter Value
Solvent CDCl3
Spectrometer Frequency 100.62
7
f1 (ppm)

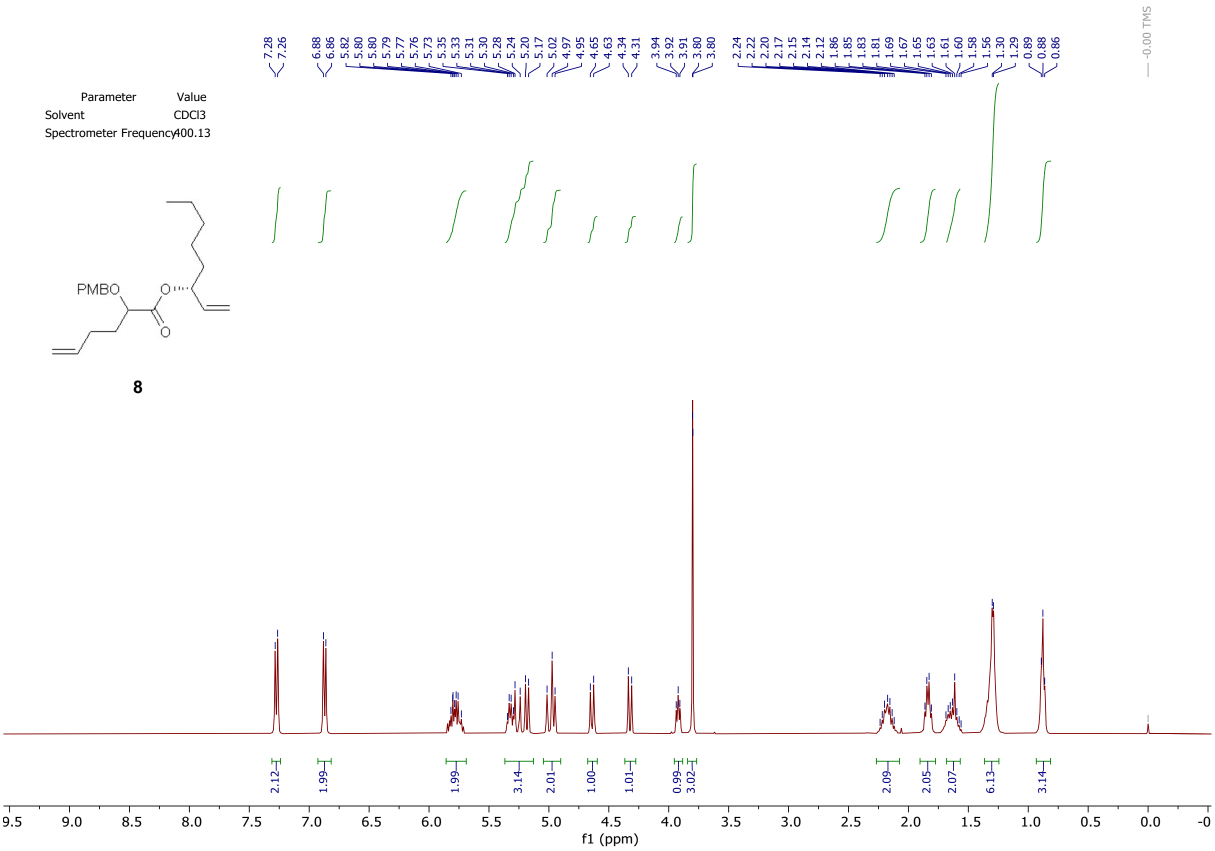
7.28
7.26
6.88
6.86
5.82
5.80
5.79
5.77
5.76
5.73
5.35
5.33
5.31
5.30
5.28
5.24
5.20
5.17
5.02
4.97
4.95
4.65
4.63
4.34
4.31
3.94
3.92
3.91
3.80
3.80
2.24
2.22
2.20
2.17
2.15
2.14
2.12
1.86
1.85
1.83
1.81
1.69
1.67
1.65
1.63
1.61
1.60
1.58
1.56
1.30
1.29
0.89
0.88
0.86
-0.00 TMS
Parameter Value
Solvent CDCl3
Spectrometer Frequency 400.13
PMBO
O
O
8
2.12
1.99
1.99
3.14
2.01
1.00
1.01
0.99
3.02
2.09
2.05
2.07
6.13
3.14
f1 (ppm)

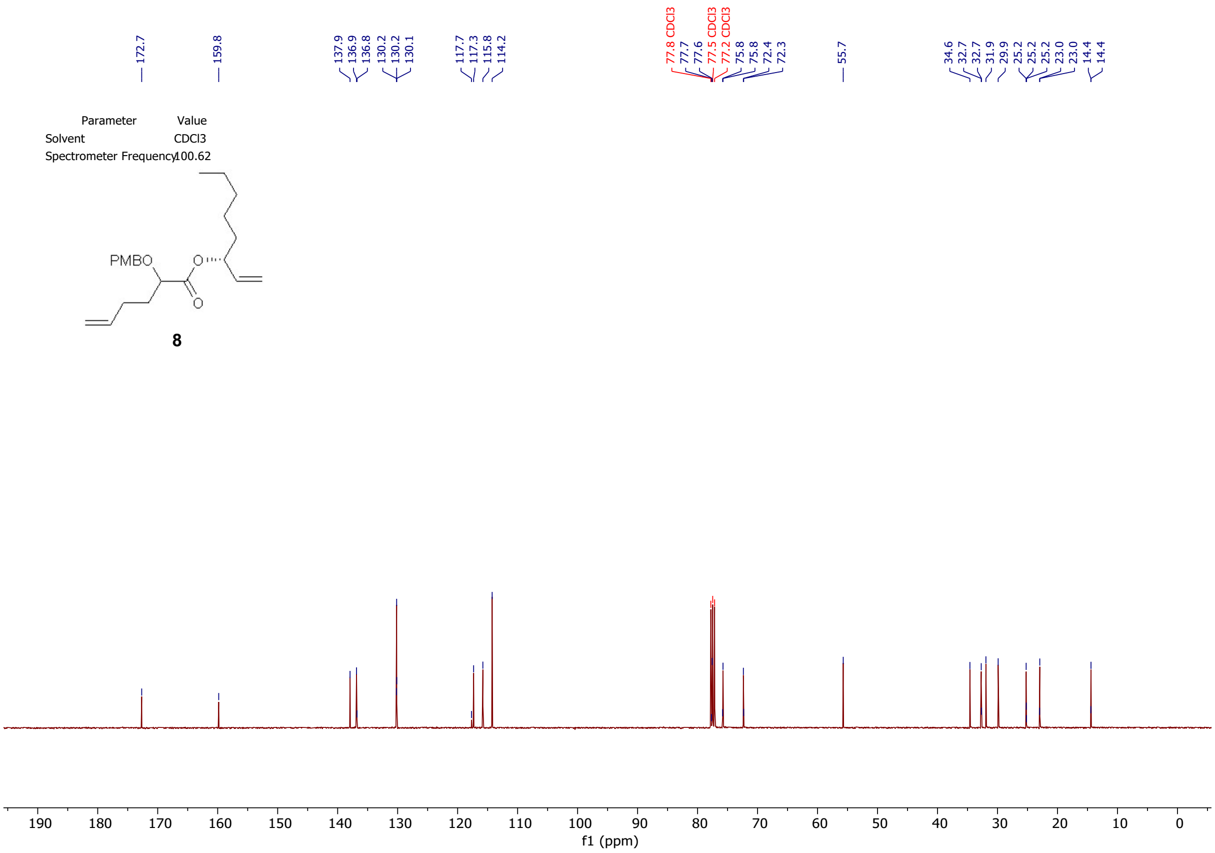
172.7
159.8
137.9
136.9
136.8
130.2
130.2
130.1
117.7
117.3
115.8
114.2
77.8 CDCl3
77.7
77.6
77.5 CDCl3
77.2 CDCl3
75.8
75.8
72.4
72.3
55.7
34.6
32.7
32.7
31.9
29.9
25.2
25.2
25.2
23.0
23.0
14.4
14.4
Parameter Value
Solvent CDCl3
Spectrometer Frequency 100.62
PMBO
O
O
8
f1 (ppm)

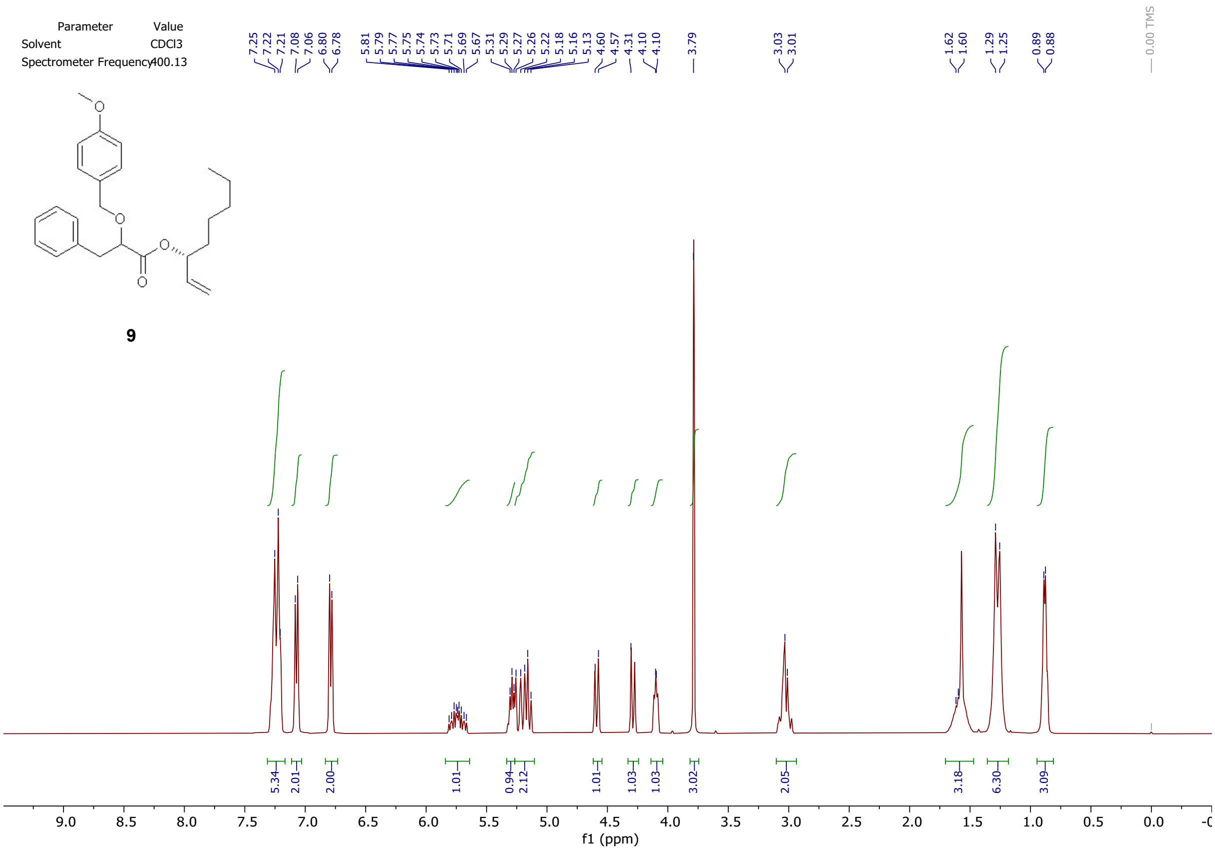
Parameter Value
Solvent CDCl3
Spectrometer Frequency 400.13
7.25 7.22 7.21 7.08 7.06 6.80 6.78
5.81 5.79 5.77 5.75 5.74 5.73 5.71 5.69 5.67 5.31 5.29 5.27 5.26 5.22 5.18 5.16 5.13
4.60 4.57 4.31 4.10 4.10
3.79
3.03 3.01
1.62 1.60 1.29 1.25 0.89 0.88
0.00 TMS
9
5.34 2.01 2.00 1.01 0.94 2.12 1.01 1.03 1.03 3.02 2.05 3.18 6.30 3.09
f1 (ppm)

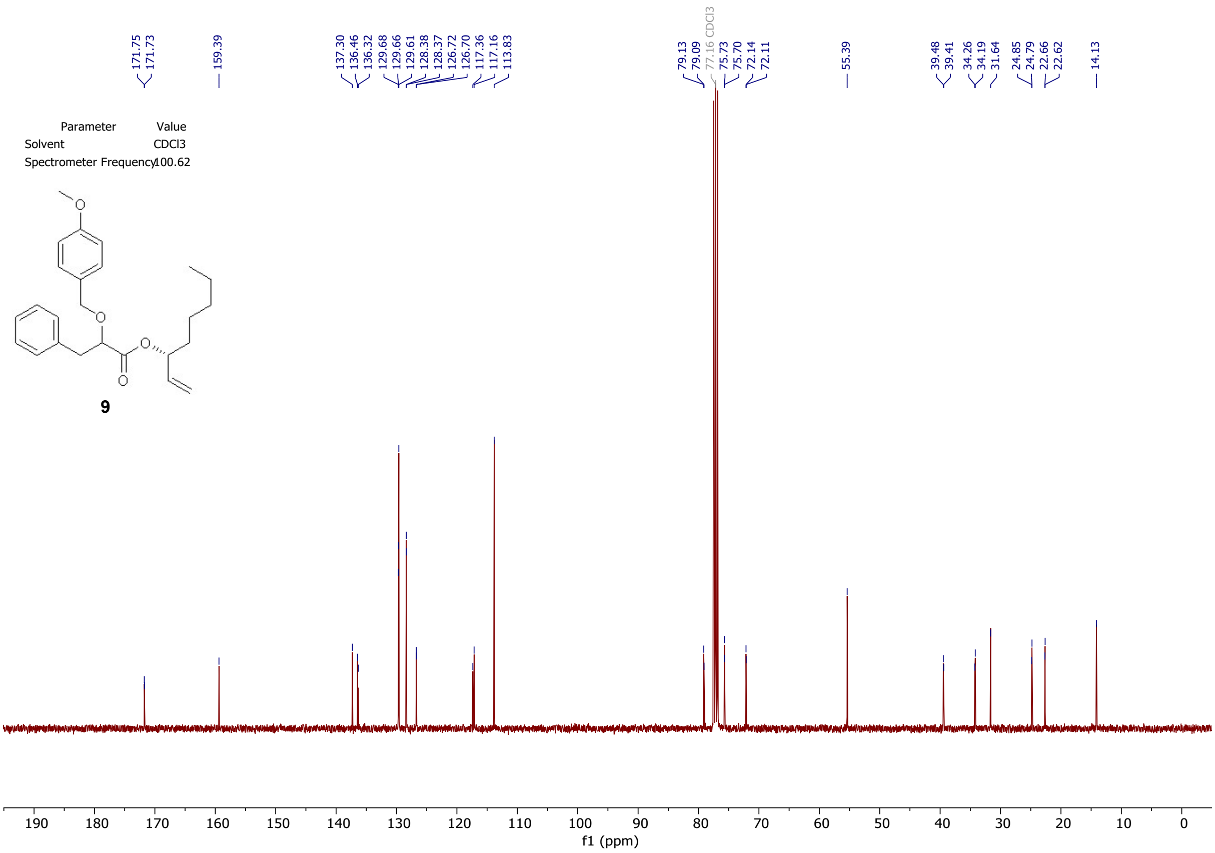
171.75 171.73
159.39
137.30 136.46 136.32 129.68 129.66 129.61 128.38 128.37 126.72 126.70 117.36 117.16 113.83
79.13 79.09 77.16 CDCl3 75.73 75.70 72.14 72.11
55.39
39.48 39.41 34.26 34.19 31.64 24.85 24.79 22.66 22.62 14.13
Parameter Value
Solvent CDCl3
Spectrometer Frequency 100.62
9
f1 (ppm)

Parameter	Value
Solvent	CDCl3
Spectrometer Frequency	400.13

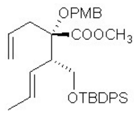

2

major diastereoisomer

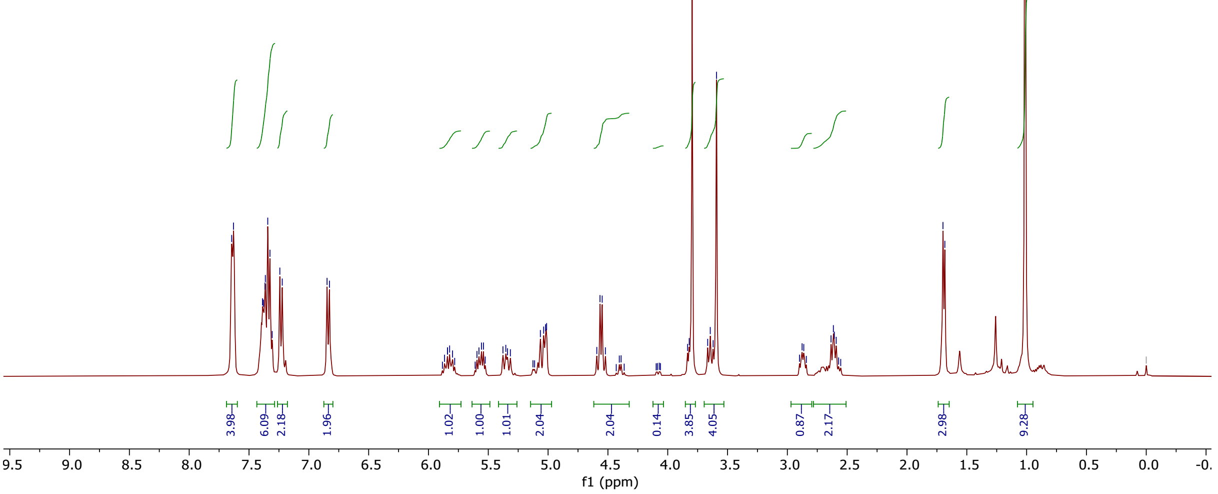

Parameter	Value
Solvent	CDCl3
Spectrometer Frequency	100.62

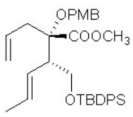

2

major diastereoisomer

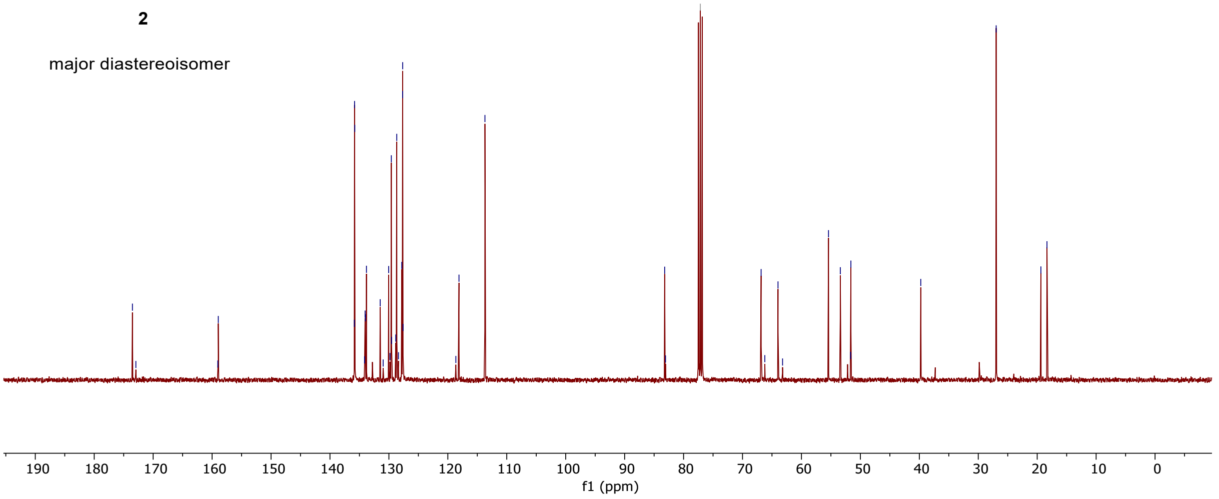

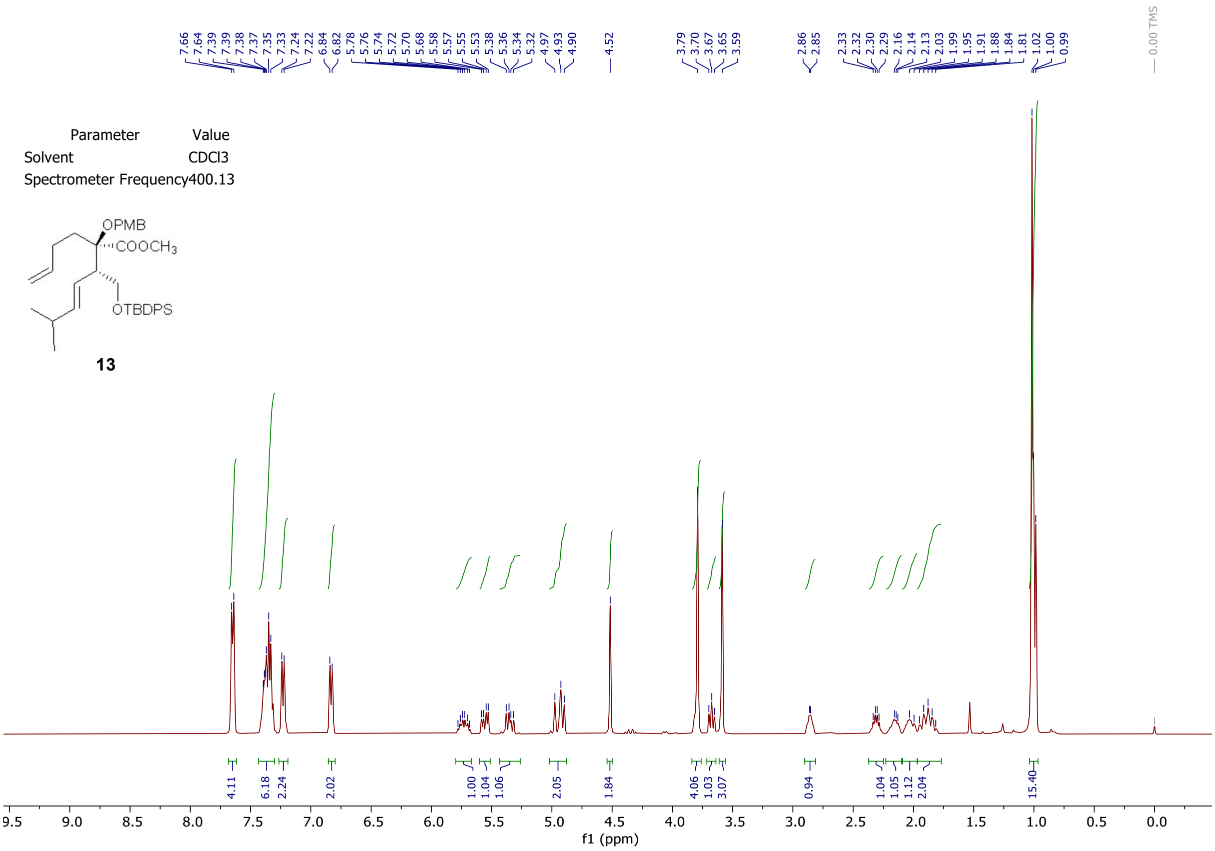
7.66 7.64 7.39 7.39 7.38 7.37 7.35 7.33 7.24 7.22 6.84 6.82 5.78 5.76 5.74 5.72 5.70 5.68 5.58 5.57 5.55 5.53 5.38 5.36 5.34 5.32 4.97 4.93 4.90 4.52 3.79 3.70 3.67 3.65 3.59 2.86 2.85 2.33 2.32 2.30 2.29 2.16 2.14 2.13 2.03 1.99 1.95 1.91 1.88 1.84 1.81 1.02 1.00 0.99
0.00 TMS
Parameter Value
Solvent CDCl3
Spectrometer Frequency400.13
OPMB
COOCH3
OTBDPS
13
4.11 6.18 2.24 2.02 1.00 1.04 1.06 2.05 1.84 4.06 1.03 3.07 0.94 1.04 1.05 1.12 2.04 15.40
9.5 9.0 8.5 8.0 7.5 7.0 6.5 6.0 5.5 5.0 4.5 4.0 3.5 3.0 2.5 2.0 1.5 1.0 0.5 0.0
f1 (ppm)

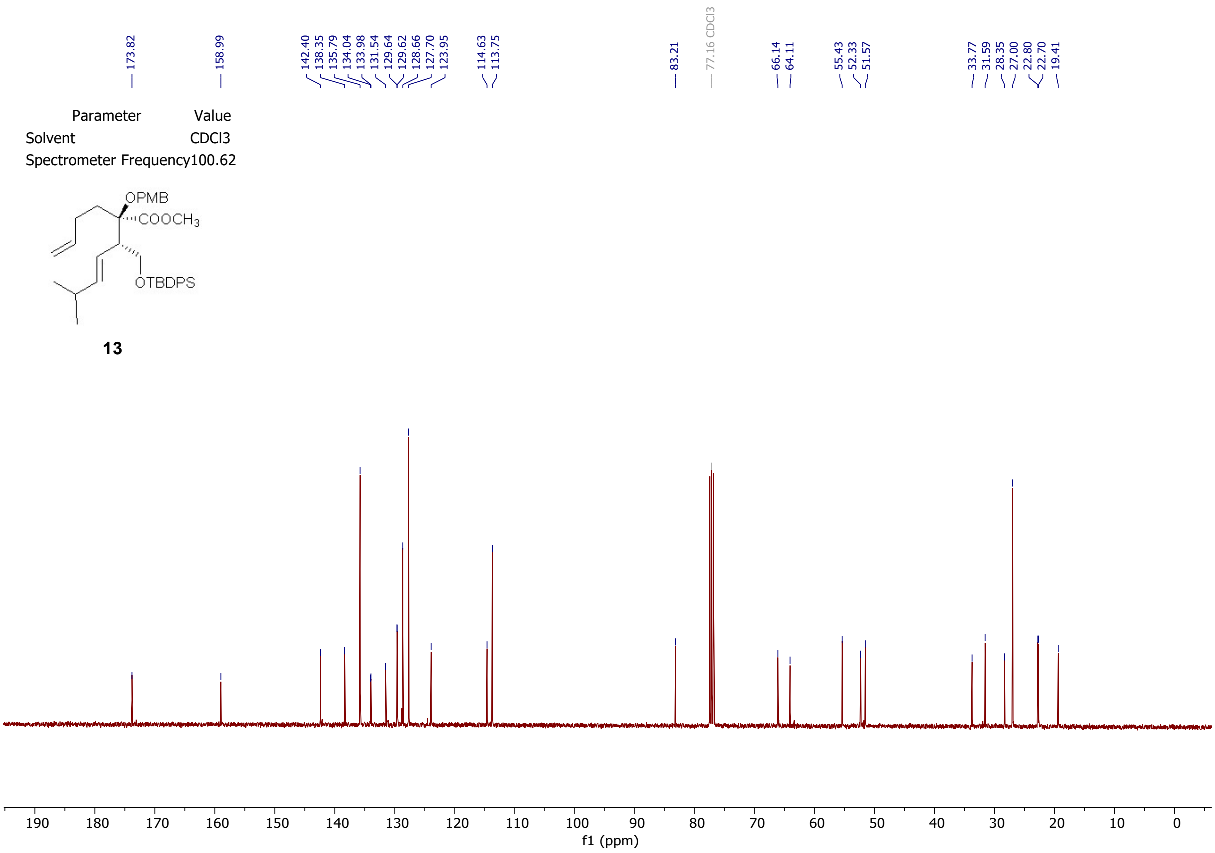
173.82 158.99 142.40 138.35 135.79 134.04 133.98 131.54 129.64 129.62 128.66 127.70 123.95 114.63 113.75 83.21
77.16 CDCl3
66.14 64.11 55.43 52.33 51.57 33.77 31.59 28.35 27.00 22.80 22.70 19.41
Parameter Value
Solvent CDCl3
Spectrometer Frequency100.62
OPMB
COOCH3
OTBDPS
13
190 180 170 160 150 140 130 120 110 100 90 80 70 60 50 40 30 20 10 0
f1 (ppm)

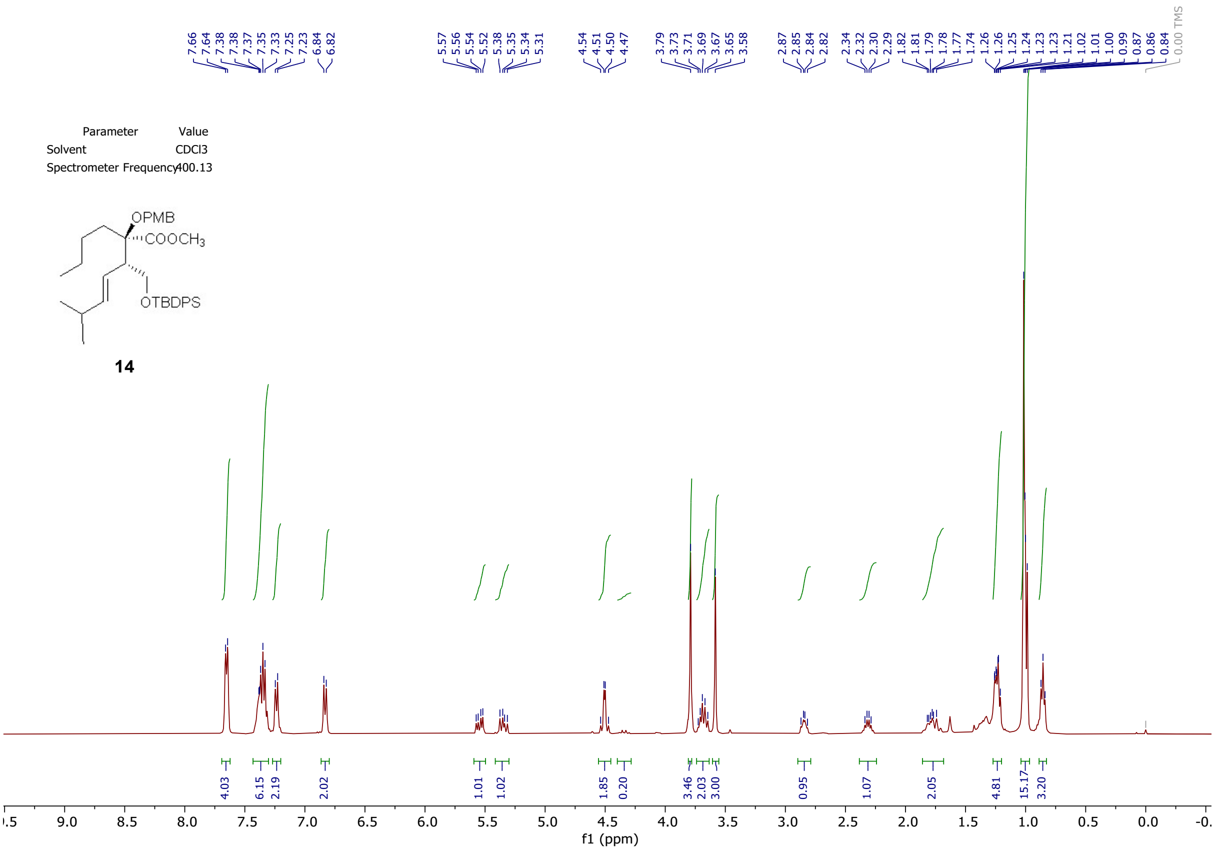
7.66 7.64 7.38 7.38 7.37 7.35 7.33 7.25 7.23 6.84 6.82 5.57 5.56 5.54 5.52 5.38 5.35 5.34 5.31 4.54 4.51 4.50 4.47 3.79 3.73 3.71 3.69 3.67 3.65 3.58 2.87 2.85 2.84 2.82 2.34 2.32 2.30 2.29 1.82 1.81 1.79 1.78 1.77 1.74 1.26 1.26 1.25 1.24 1.23 1.23 1.21 1.02 1.01 1.00 0.99 0.87 0.86 0.84
0.00 TMS
Parameter Value
Solvent CDCl3
Spectrometer Frequency 400.13
OPMB
COOCH3
OTBDPS
14
4.03 6.15 2.19 2.02 1.01 1.02 1.85 0.20 3.46 2.03 3.00 0.95 1.07 2.05 4.81 15.17 3.20
f1 (ppm)

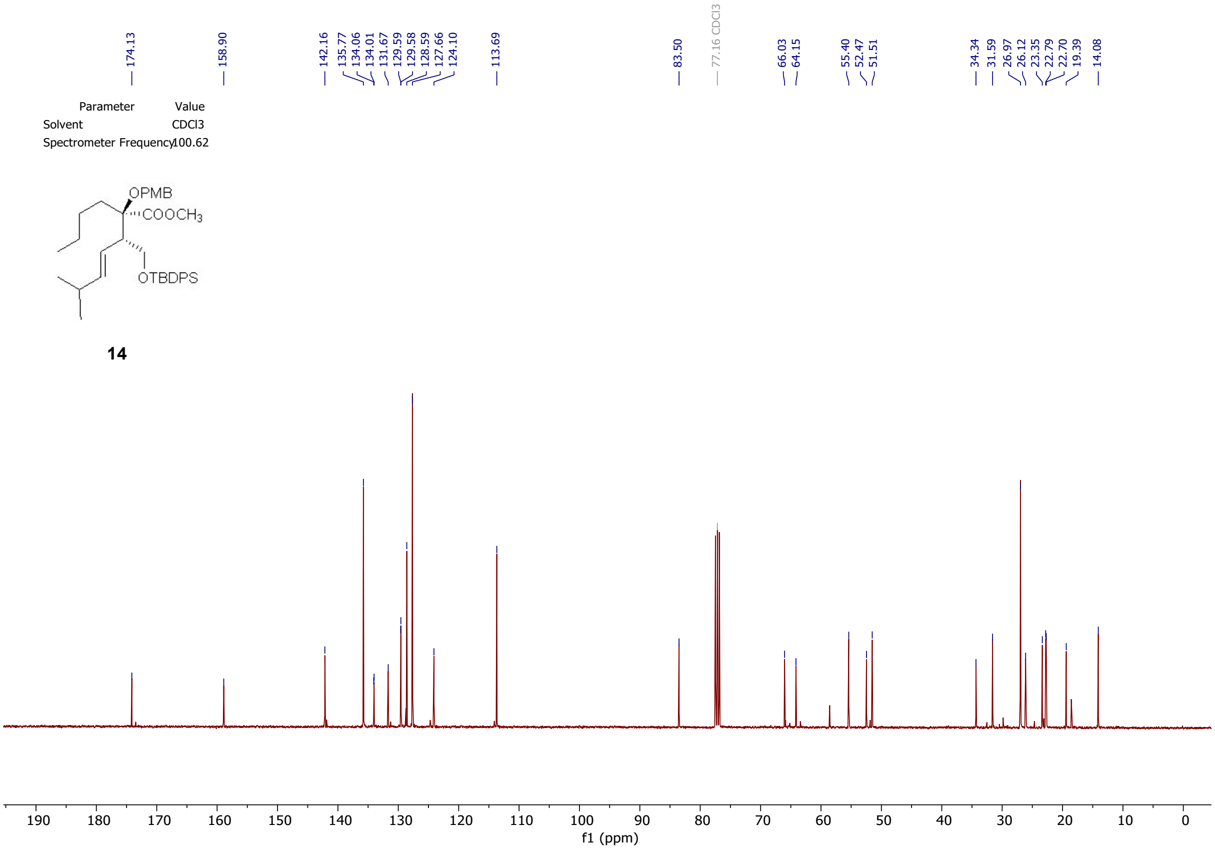
174.13 158.90 142.16 135.77 134.06 134.01 131.67 129.59 129.58 128.59 127.66 124.10 113.69 83.50
77.16 CDCl3
66.03 64.15 55.40 52.47 51.51 34.34 31.59 26.97 26.12 23.35 22.79 22.70 19.39 14.08
Parameter Value
Solvent CDCl3
Spectrometer Frequency 100.62
OPMB
COOCH3
OTBDPS
14
f1 (ppm)

Parameter	Value
Solvent	CDCl3
Spectrometer Frequency	400.13

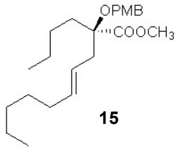

15

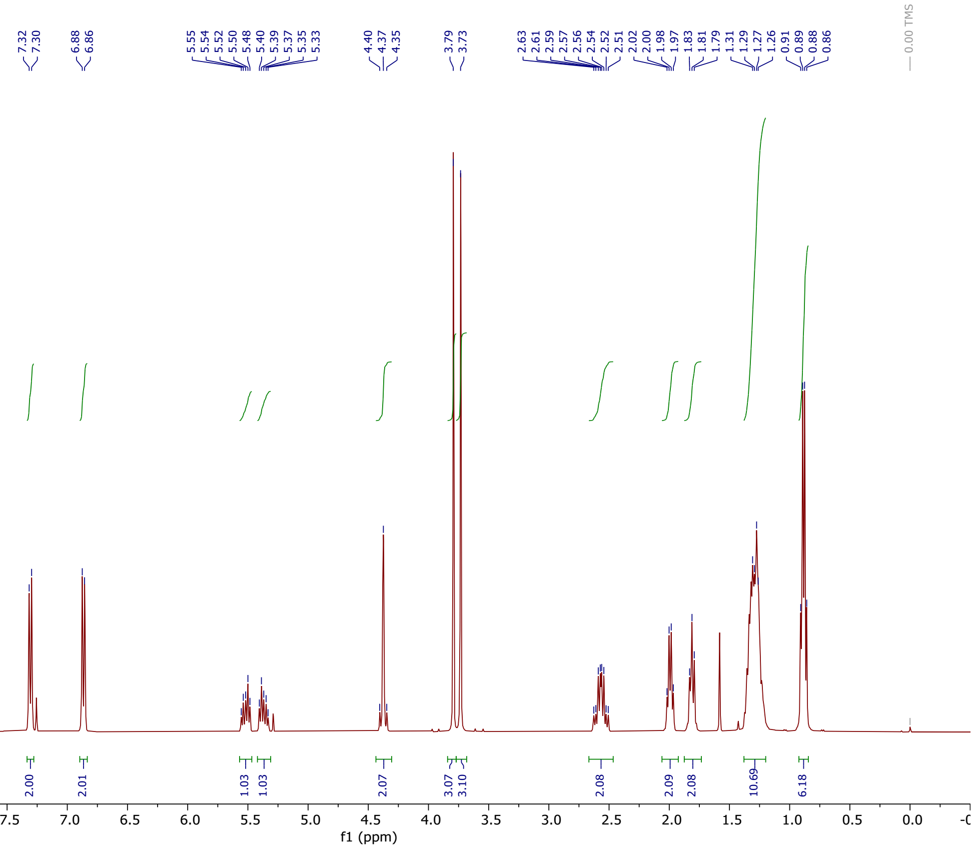

Parameter	Value
Solvent	CDCl3
Spectrometer Frequency	100.62

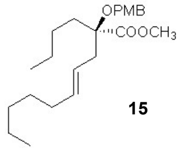

15

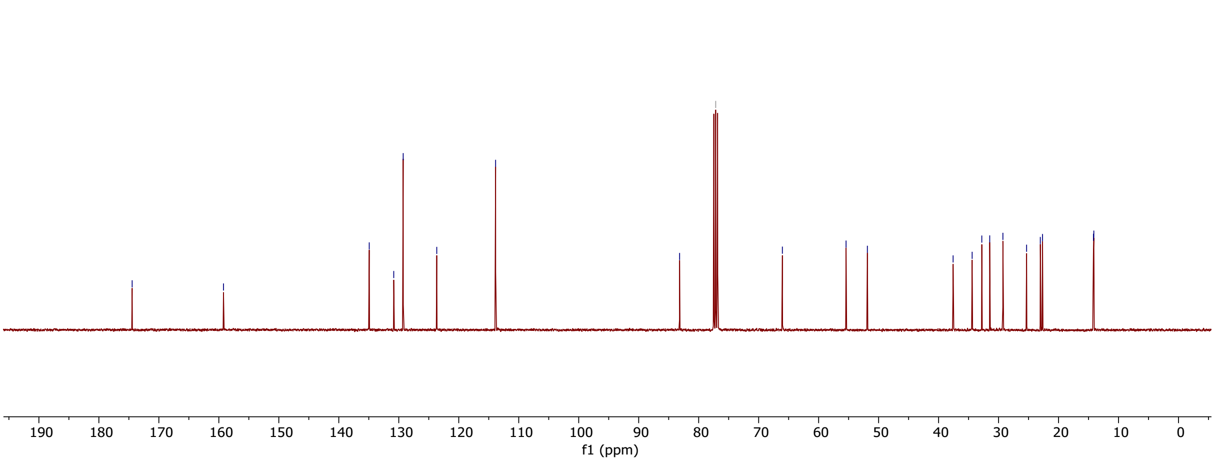

Parameter	Value
Solvent	CDCl3
Spectrometer Frequency	400.13

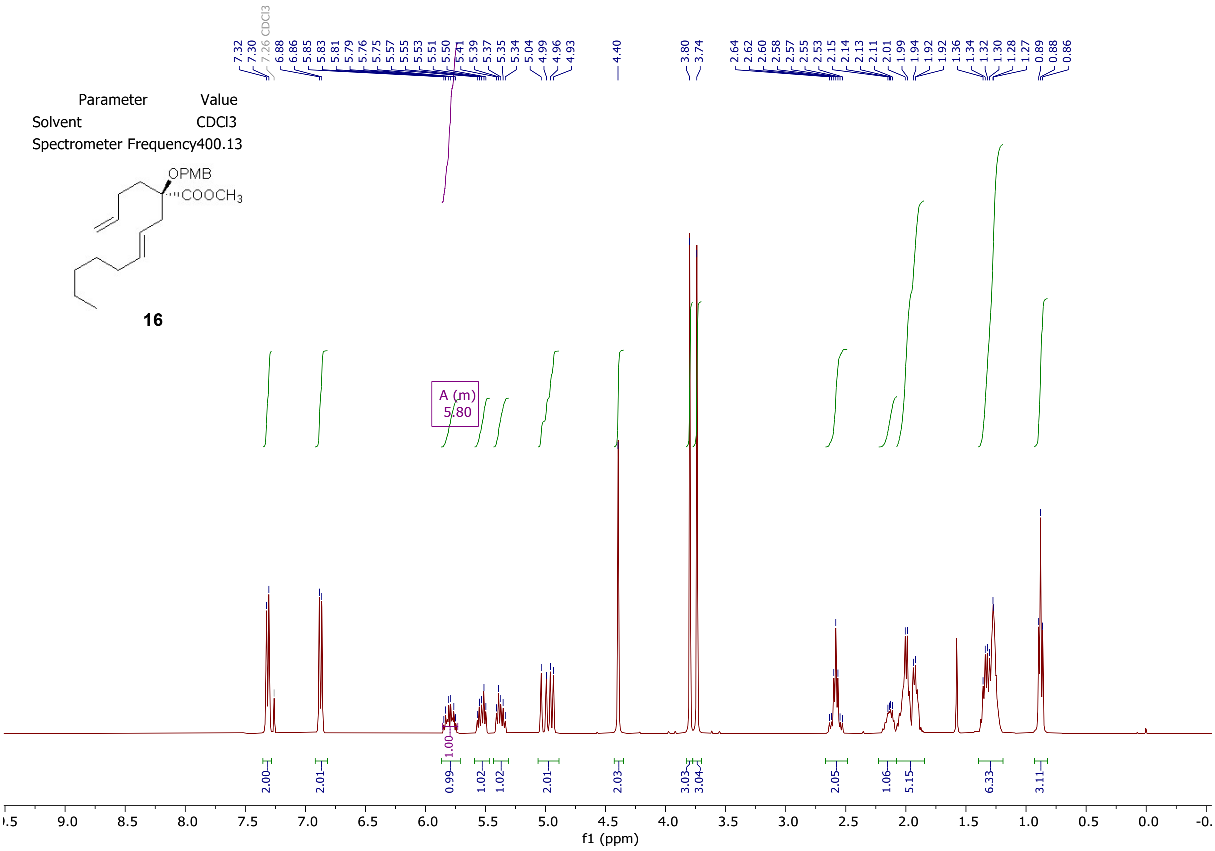

16

Parameter	Value
Solvent	CDCl3
Spectrometer Frequency	100.62

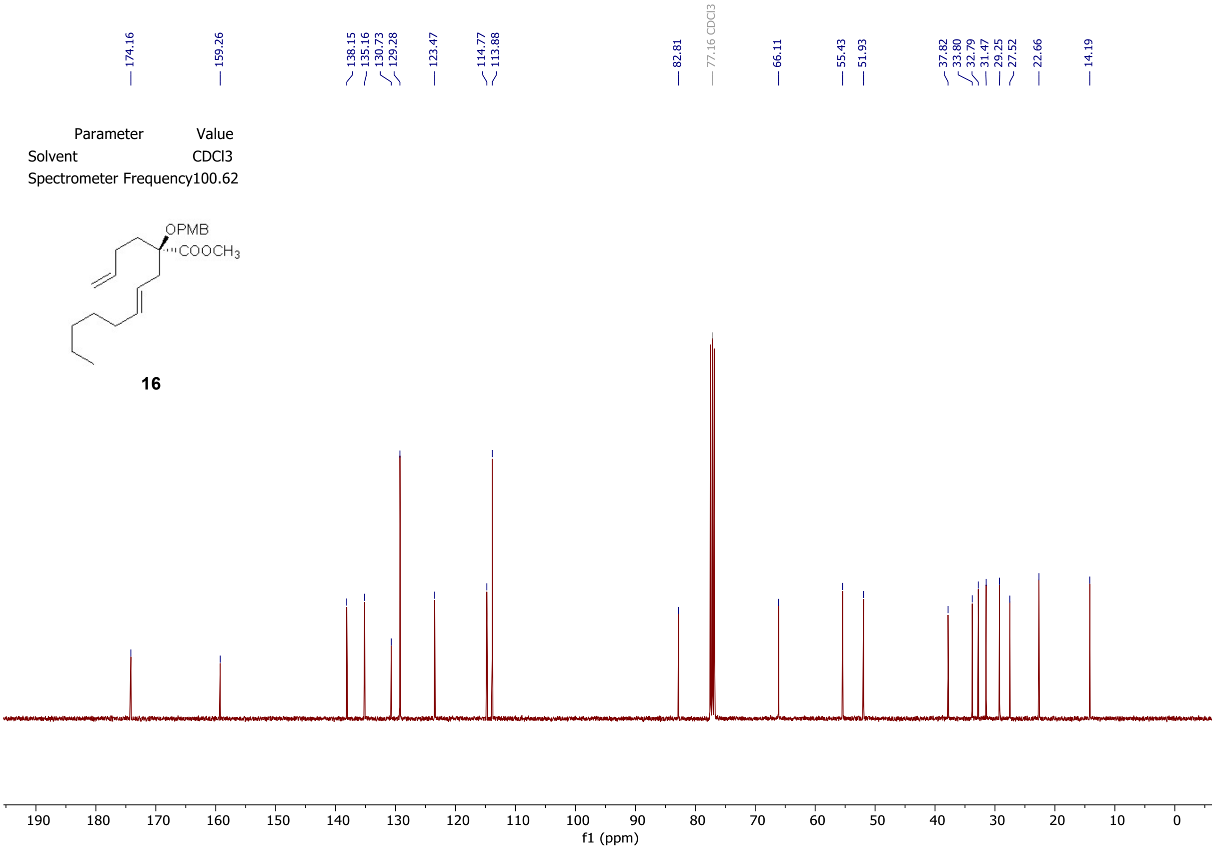

16

Parameter	Value
Solvent	CDCl3
Spectrometer Frequency	400.13

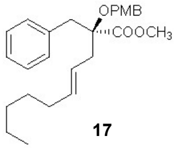

17

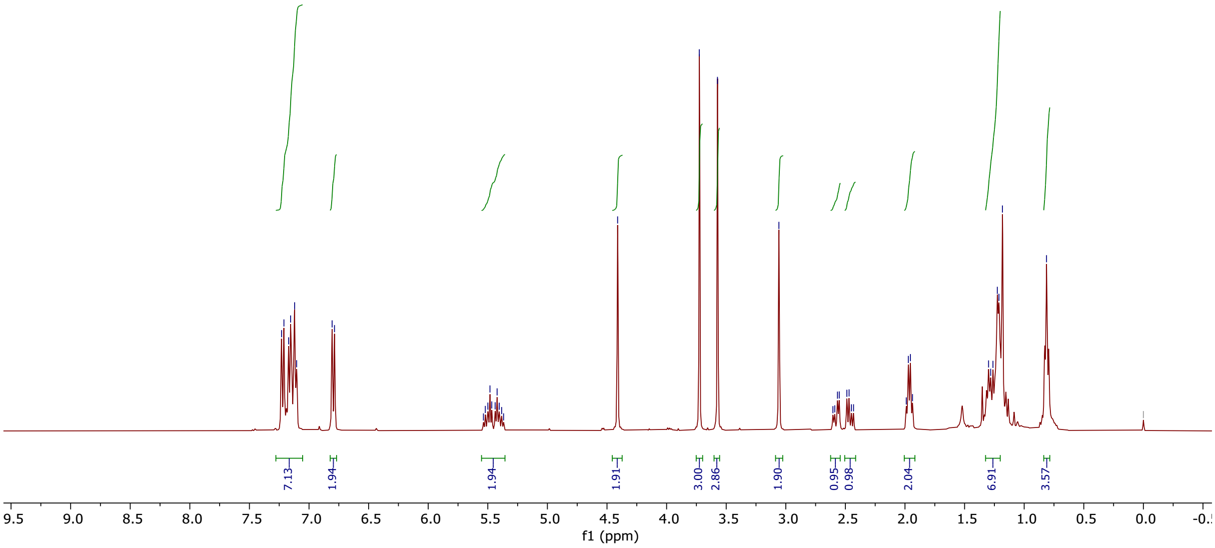

Parameter	Value
Solvent	CDCl3
Spectrometer Frequency	100.62

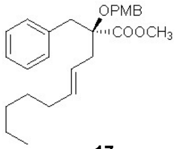

17

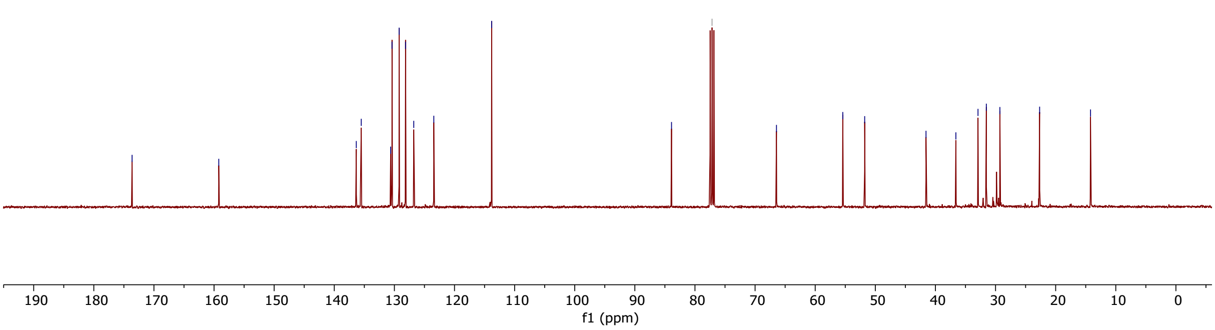

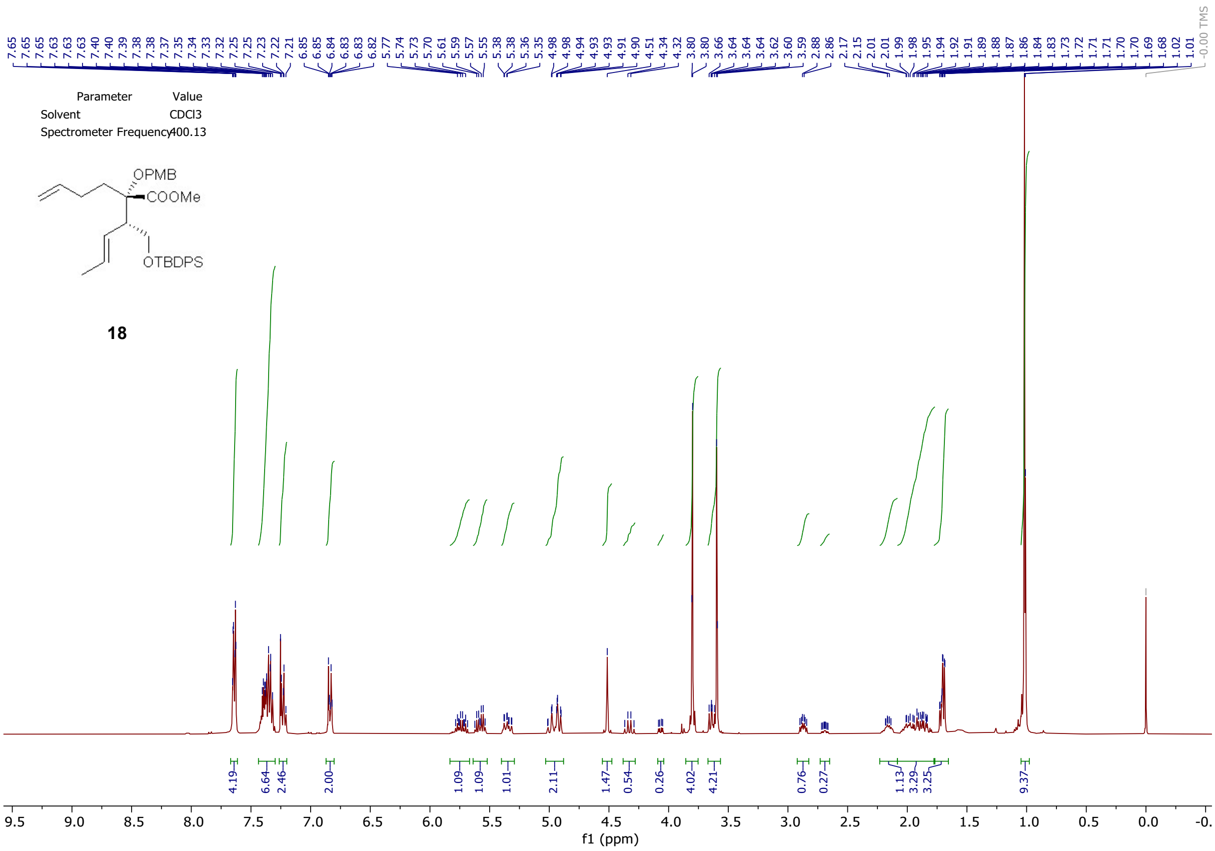
7.65 7.65 7.63 7.63 7.63 7.40 7.40 7.39 7.38 7.38 7.37 7.35 7.34 7.33 7.32 7.25 7.25 7.23 7.22 7.21 6.85 6.85 6.84 6.83 6.83 6.82 5.77 5.74 5.73 5.70 5.61 5.59 5.57 5.55 5.38 5.38 5.36 5.35 4.98 4.98 4.94 4.93 4.93 4.91 4.90 4.51 4.34 4.32 3.80 3.80 3.66 3.64 3.64 3.64 3.62 3.60 3.59 2.88 2.86 2.17 2.15 2.01 2.01 1.99 1.98 1.95 1.94 1.92 1.91 1.89 1.88 1.87 1.86 1.84 1.83 1.73 1.72 1.71 1.71 1.70 1.70 1.69 1.68 1.02 1.01
-0.00 TMS
Parameter Value
Solvent CDCl3
Spectrometer Frequency 400.13
OPMB
COOMe
OTBDPS
18
4.19 6.64 2.46 2.00 1.09 1.09 1.01 2.11 1.47 0.54 0.26 4.02 4.21 0.76 0.27 1.13 3.29 3.25 9.37
9.5 9.0 8.5 8.0 7.5 7.0 6.5 6.0 5.5 5.0 4.5 4.0 3.5 3.0 2.5 2.0 1.5 1.0 0.5 0.0 -0.
f1 (ppm)

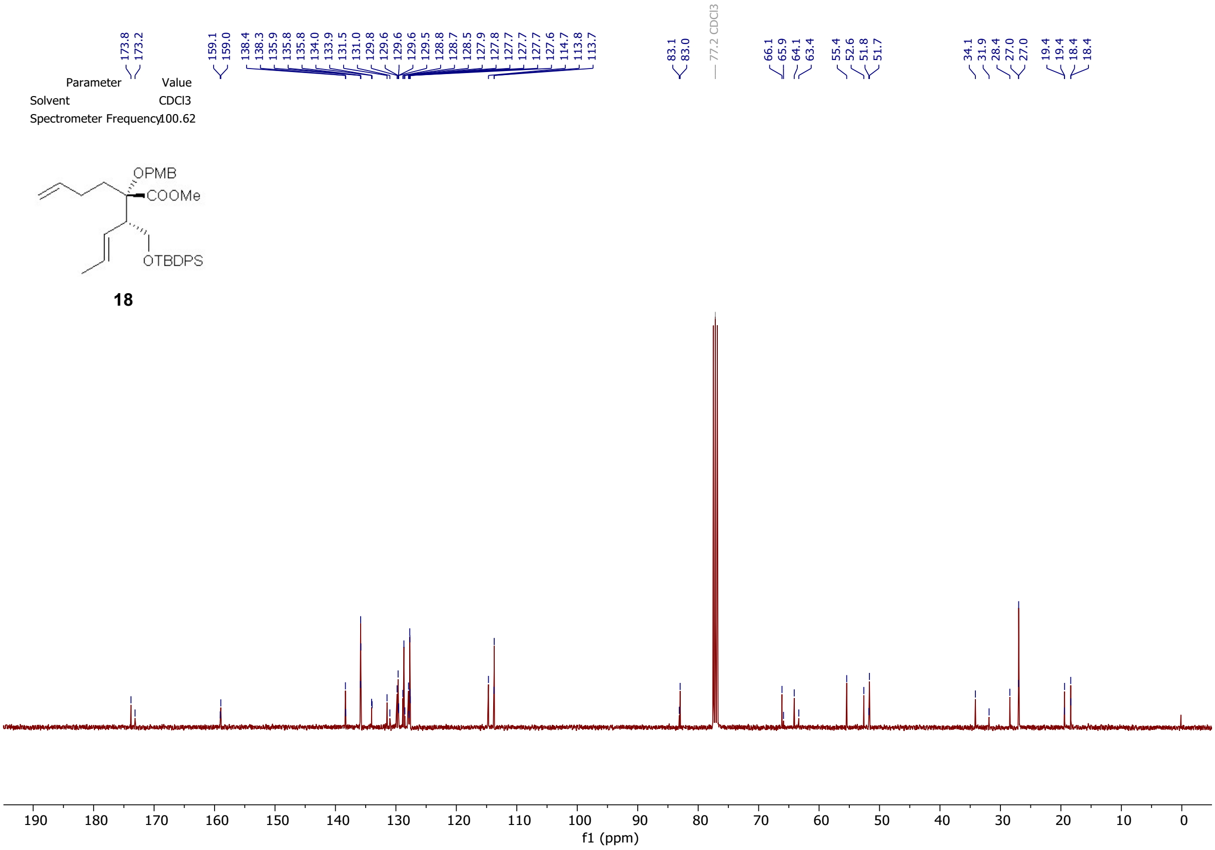
173.8 173.2 159.1 159.0 138.4 138.3 135.9 135.8 134.0 133.9 131.5 131.0 129.8 129.6 129.6 129.5 128.8 128.7 128.5 127.9 127.8 127.7 127.7 127.7 127.6 114.7 113.8 113.7 83.1 83.0
77.2 CDCl3
66.1 65.9 64.1 63.4 55.4 52.6 51.8 51.7 34.1 31.9 28.4 27.0 27.0 19.4 19.4 18.4 18.4
Parameter Value
Solvent CDCl3
Spectrometer Frequency 100.62
OPMB
COOMe
OTBDPS
18
190 180 170 160 150 140 130 120 110 100 90 80 70 60 50 40 30 20 10 0
f1 (ppm)
